# Guanidine‐Derived Polymeric Nanoinhibitors Target the Lysosomal V‐ATPase and Activate AMPK Pathway to Ameliorate Liver Lipid Accumulation

**DOI:** 10.1002/advs.202408906

**Published:** 2024-11-05

**Authors:** Yunfei Zhao, Ke Hu, Fangliang Wang, Lulu Zhao, Yu Su, Jun Chen, Gang Zou, Liming Yang, Li Wei, Mengjiao Deng, Yunyu He, Ping Wang, Xiong Z Ruan, Yaxi Chen, Chao Yu

**Affiliations:** ^1^ Chongqing Medical University College of Pharmacy Chongqing Key Laboratory for Pharmaceutical Metabolism Research Chongqing Pharmacodynamic Evaluation Engineering Technology Research Center Chongqing 400016 P. R. China; ^2^ Centre for Lipid Research & Chongqing Key Laboratory of Metabolism on Lipid and Glucose Key Laboratory of Molecular Biology for Infectious Diseases (Ministry of Education) Institute for Viral Hepatitis Department of Infectious Diseases the Second Affiliated Hospital Chongqing Medical University Chongqing 400016 P. R. China

**Keywords:** AMPK, liver lipid accumulation, lysosome, polyguanide nanoinhibitor (PGNIs), V‐ATPase

## Abstract

Current research efforts in polymer and nanotechnology applications are primarily focused on cargo delivery to enhance the therapeutic index, with limited attention being paid to self‐molecularly targeted nanoparticles, which may also exhibit significant therapeutic potential. Long‐term and anomalous lipid accumulation in the liver is a highly relevant factor contributing to liver diseases. However, the development of the reliable medications and their pharmacological mechanisms remain insufficient. Herein, a polyguanide nanoinhibitors (PGNI) depot is constructed by copolymerizing biguanide derivatives in different proportions onto prepolymers. The nanoinhibitors for their ability to ameliorate lipid accumulation in vitro and in vivo is screened, and subsequently demonstrated that covalently polymeric guanidine chains exhibit superior efficacy in ameliorating hepatic lipid accumulation via heterogeneous mechanisms compared to small‐molecule guanidine. It is found that PGNIs stabilize guanidine metabolism in the liver, preferably for biosafety. More importantly, PGNI is ingested and localized in hepatocyte lysosomes and is locked to interact with vesicular adenosine triphosphatase (V‐ATPase) on lysosomes, leading to the inhibition of V‐ATPase and lysosomal acidification, thereby activating the AMPK pathway, reducing fatty acid synthesis, and enhancing lipolysis and fatty acid oxidation. These results imply that polymer‐formed nanoparticles can serve as targeted inhibitors, offering a novel approach for therapeutic applications.

## Graphical abstract

In this work, a polyguanide nanoinhibitors (PGNI) depot is constructed by copolymerizing biguanide derivatives in different proportions onto prepolymers. The nanoinhibitors for their ability to ameliorate lipid accumulation in vitro and in vivo is screened, and subsequently demonstrated that covalently polymeric guanidine chains exhibit superior efficacy in ameliorating hepatic lipid accumulation via heterogeneous mechanisms compared to small‐molecule guanidine. More importantly, PGNI is ingested and localized in hepatocyte lysosomes and is locked to interact with vesicular adenosine triphosphatase (V‐ATPase) on lysosomes, leading to the inhibition of V‐ATPase and lysosomal acidification, thereby activating the AMPK pathway, reducing fatty acid synthesis, and enhancing lipolysis and fatty acid oxidation. These results imply that polymer‐formed nanoparticles can serve as targeted inhibitors, offering a novel approach for therapeutic applications.

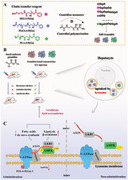

## 1 Introduction

Nonalcoholic fatty liver disease (NAFLD), the most common chronic liver disease, affects one‐quarter of the global population worldwide and is associated with metabolic syndrome and obesity, whose incidence is increasing.^[^ ^1^, ^2^, ^3^ ^]^ Reducing hepatic lipid deposition is the key to inhibition of NAFLD progression.^[^ ^4^ ^]^ However, the use of medication to ameliorate of hepatic lipid deposition is limit.^[^ ^5^, ^6^, ^7^ ^]^ The reversal of excessive hepatic lipid deposition remains a challenge in drug design.

At doses far greater than those tolerable in actual use (300 mg kg^−1^ in mice, converted to an adult dose of 2.7 g kg^−1^), small‐molecule guanidine compounds, with metformin as a typical representative, have demonstrated the potential to improve intracellular lipid deposition to varying degrees in hepatocytes and animal experiments,^[^ ^8^, ^9^, ^10^, ^11^ ^]^ however, the clinical data on their pharmacodynamics are still highly controversial.^[^ ^12^, ^13^ ^]^ Notably, the pharmacological effects of this class of drugs depend on the guanidine group.^[^ ^14^ ^]^ However, hydrophilic guanidineresults in low drug bioavailability. For example, many studies have shown that metformin uptake and elimination occur at similar rates in the liver via bidirectional transport.^[^ ^15^, ^16^ ^]^ This factor may limit the effective concentration of small‐molecule guanidine compounds in the liver and explain the intolerable dose of metformin for NAFLD amelioration and the clinical dispute mentioned above.

ngly, we previously discovered that polymerized guanidine has more effective weight control and reduced liver lipid deposition in vivo than small‐molecule guanidine compounds.^[^ ^17^ ^]^ We speculate the unique long‐chain structure, molecular weight, and intermolecular forces of polymers cause the guanidine groups to exhibit entirely different quantitative and qualitative changes in terms of structural characteristics and biological effects. First, in terms of structure, each molecule of polymeric guanidine contains more guanidine groups. These additional guanidine groups may render it more efficacious compared to small molecular guanidine. Second, with the increase of the molecular weight of the polymeric compound, the molecular chain becomes longer and more complex, and the intermolecular forces will also change. The intermolecular forces between polymerized guanidine ameliorate small‐molecule guanidine compounds excessive water solubility to a certain extent while forming nanoparticles in the aqueous phase. Leveraging the natural targeting advantage of nanoparticles to the liver, polymeric guanidine has a longer retention time in the liver, enhancing the bioavailability of guanidine in the liver. However, these polymeric compounds also have complex characteristics. The molecular weight (Mw), architecture, assembly structure, and subcellular location affect the pharmacodynamics of polymeric drugs.^[^ ^18^ ^]^ To solve this problem, in this study, a methacrylic biguanide derivative N‐(2‐(3‐carbamimidoylguanidino)ethyl)methacrylamide (GuC) was designed and synthesized, and controllable copolymerization was performed on three universal medically degradable polymers (polyethylene glycol (PEG), polylactic‐co‐glycolic acid (PLGA), and polylactic acid (PLA)) by reversible addition‐fragmentation chain transfer (RAFT) using a modified prepolymer in different proportions to fabricate 15 guanidine‐derived copolymers, followed by their dynamic self‐assembly to spherical nanoinhibitors (including PEG‐***b***‐P(Gu)1‐5, PLGA‐***b***‐P(Gu)1‐5, PLA‐***b***‐P(Gu)1‐5).

To identify the druggable interaction site, we focused on the vesicular adenosine triphosphatase (V‐ATPase) protein in lysosomes for three reasons: First, most of the macromolecules are mainly ingested into cells through endocytosis,^[^ ^19^, ^20^, ^21^ ^]^ similarly, PGNIs is localized to lysosomes; and the pathway related to phagocytosis in the liver is significantly activated after administration. Thus, we suspected that PGNIs mainly interacts with lysosomal related proteins for pharmacological effects, which is differ from the fact that the small molecule guanidine mainly enters hepatocytes through organic cation transporters 1 or 3 (OCT1, OCT3).^[^ ^22^, ^23^, ^24^ ^]^ Second, recent studies have found an intimate relationship between V‐ATPase and guanidine groups.^[^ ^25^ ^]^ Specifically, low doses of metformin target the lysosomal membrane protein presenilin enhancer 2 (PEN2), allowing PEN2 to gain affinity for the V‐ATPase subunit ATP6AP1 and form a complex. This process leads to the inhibition of V‐ATPase, which in turn changes the conformation of V‐ATPase and Ragulator (LAMTOR) and then initiates the recruitment of the axis protein (AXIN) and liver kinase B1 (LKB1) to the outer lysosomal leaflets, where they bind to V‐ATPase/LAMTOR. Finally, AMP‐activated protein kinase (AMPK) is activated.^[^ ^25^ ^]^ Activated AMPK inhibits de novo fatty acid synthesis, and promotes lipolysis and fatty acid oxidation, ultimately reducing cellular lipid accumulation. Third, in our previous study, carriers containing guanidine groups showed evidence of a lysosomal escape effect to a certain degree.^[^ ^17^ ^]^ Notably, this effect is often closely related to the consumed H^+^ and the increased pH of lysosome. V‐ATPase is the molecular motor protein that controls the acidic environment of lysosomes. Inhibition of V‐ATPase will cause a decrease in the ability of the lysosomal pump to transport H^+^, leading to an increase in pH.

**Figure 1:**
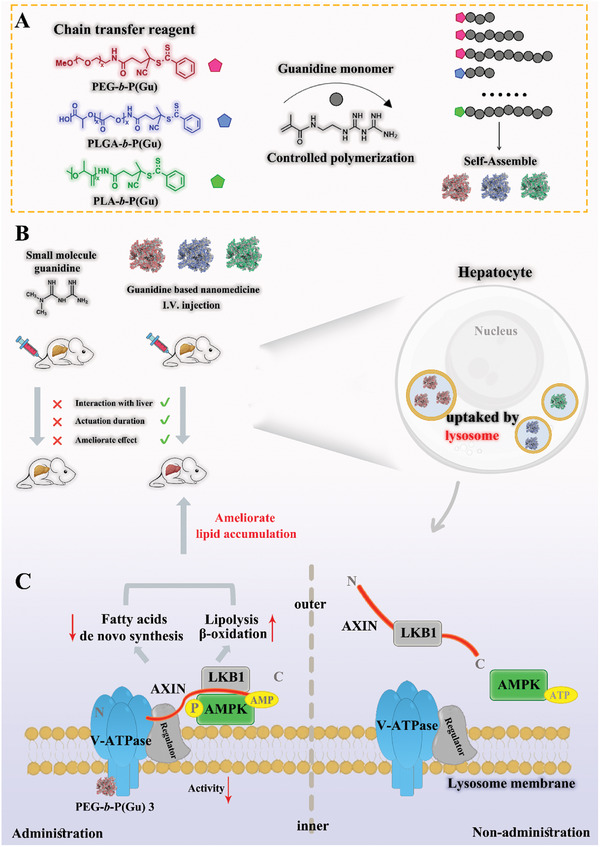
Diagram of the synthesis of polyguanidine nanomedicine for ameliorating liver accumulation. A) The synthesis and self‐assembly process of PEG‐***b***‐P(Gu), PLGA‐***b***‐P(Gu), and PLA‐***b***‐P(Gu). B) Polyguanidine nanomedicine ameliorates hepatic lipid accumulation in mice and its lysosomal uptake in hepatocytes. C) PEG‐***b***‐P(Gu) accumulates at the lysosome and further triggers AMPK activation. PEG‐***b***‐P(Gu) binds to the inner leaflet of the V‐ATPase and inhibits its activity, followed by AXIN and LKB1 being recruited to the V‐ATPase and Regulator (LAMTOR) for inducing AMPK phosphorylation and subsequently promoting lipolysis and reducing the fatty acid de novo synthesis.

In this study, we fabricated a series of PGNI depots (**Figure** **1**A) by RAFT polymerization. By controlling the quantity of guanidine, it was copolymerized onto PEG/PLGA/PLA‐modified 4‐cyano‐4‐(phenyl thioformyl sulfur) valeric acid (PEG‐CPADN/PLGA‐CPADN/PLA‐CPADN), which self‐assembled into nanoparticles. As depicted in Figure 1B, upon administration, the PGNIs naturally accumulate in the liver and are taken up by hepatocytes. After entering the lysosome, the PGNIs interact with and target the V_0_ region of V‐ATPase in the inner leaflets of the lysosome. This interaction leads to an inhibition of V‐ATPase activity, resulting in the formation of V‐ATPase‐Ragulator‐AXIN/LKB1‐AMPK complex and activation of the AMPK pathway. Furthermore, activated AMPK inhibits fatty acid de novo synthesis, and promotes lipolysis and β‐oxidation, ultimately ameliorating lipid accumulation in the liver (Figure 1C).

## 2 Results and Discussion

### 2.1 Macromolecule Design and Synthesis

**Figure 2:**
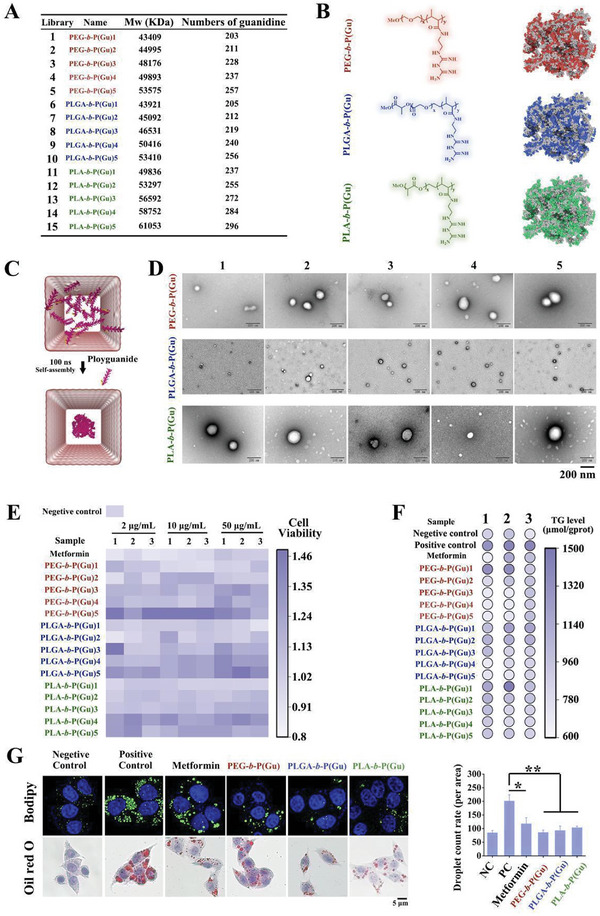
Basic characterization and lipid deposition lowering effect of polyguanidine nanomedicine in vitro. A) Average molecular weights (Mw) of a library of 15 polyguanidine nanomedicines synthesized by different feed ratios and the theoretical values of the guanidine chains calculated by Mw. B) Chemical formulas of PEG‐***b***‐P(Gu), PLGA‐***b***‐P(Gu), and PLA‐***b***‐P(Gu) and their 3D self‐assembly schematic diagrams. C) Molecular dynamics simulation of polyguanidine conformation change in 100 ns and D) TEM image of the polyguanidine nanomedicine library. Scale bar: 200 nm. E) Cell viability of HepG2 cells treated with metformin, PEG‐***b***‐P(Gu)1‐5, PLGA‐***b***‐P(Gu)1‐5, and PLA‐***b***‐P(Gu)1‐5 in different concentrations. F) Triglyceride levels in HepG2 cells, including negative control, positive control (palmitic acid loading), metformin, PEG‐***b***‐P(Gu)1‐5, PLGA‐***b***‐P(Gu)1‐5, and PLA‐***b***‐P(Gu)1‐5. G) Bodipy and Oil Red O staining and lipid content rate of HepG2 cells treated with PEG‐***b***‐P(Gu)1‐5, PLGA‐***b***‐P(Gu)1‐5, and PLA‐***b***‐P(Gu)1‐5. Scale bar: 5 µm; ^*^ *P* < 0.05; ^**^ *P* < 0.01; bars represent the mean ± SD (n = 3). The P‐values are calculated using one‐way ANOVA with Bonferroni correction.

As shown in Figure S1 (Supporting Information), PEG‐CPADN (compound 2), PLGA‐CPADN (compound 5), and PLA‐CPADN (compound 7) were synthesized. The ‐CONH‐ bonds of the compound structures were confirmed by infrared spectroscopy (Figures S2–S4, Supporting Information). Subsequent RAFT copolymerizations at different molar ratios (Figure S5, Supporting Information) of N‐(2‐(3‐carbamimidoylguanidino)ethyl)methacrylamide (GuC, compound 3) monomers afforded PEG‐***b***‐P(Gu)1‐5 (compound 4), PLGA‐***b***‐P(Gu)1‐5 (compound 6), and PLA‐***b***‐P(Gu)1‐5 (compound 8) using PEG‐CPADN, PLGA‐CPADN, PLA‐CPADN, and azodiisobutyronitrile (AIBN) as macroinitiators. Further, ^1^H nuclear magnetic resonance (^1^H‐NMR, Figures S6–S8, Supporting Information) and gel permeation chromatography (GPC, **Figure** **2**B; Figure S9, Supporting Information) revealed an increase in the proportion of guanidine monomers and the average molecular weight (Mw) of the polymers. The theoretical number of guanidine monomers was calculated based on the molecular weight (Figure 2A). Owing to the positively charged nature of guanidine, its zeta potential, which was also used to characterize the degree of guanidine polymerization (Figure S10, Supporting Information), increased from 15 to 25 mV as the number of guanidine monomers increased.

### 2.2 Molecular Dynamics Simulation and Characterization of PGNIs

To explore the dynamic behavior of polyguanidines in the aqueous phase, molecular dynamics (MD) was used to simulate the formation processes of PGNIs. The formulae and self‐assembled conformations are shown in Figure 2B. Furthermore, cluster analysis was performed on the simulated trajectories ranging from 80 to 100 ns using the GROMACS cluster module. As shown in Figure 2C, the polyguanidines interacted with each other and formed spherical nanoclusters. To further investigate the driving forces during the interaction and aggregation processes of the system, the interactions between the polyguanidines were analyzed. As shown in Figure S11A (Supporting Information), the N atoms on the polyguanidines formed a large number of hydrogen bonds with other surrounding polyguanidines. The number of hydrogen bonds (Figure S11B, Supporting Information), root‐mean‐square deviation (RMSD; Figure S11C, Supporting Information), and solvent‐accessible surface area (SASA; Figure S11D, Supporting Information) tended to stabilize after 60 ns, indicating stable aggregation of the polyguanidines. Next, the interaction energies between the polyguanidines in the system were calculated. Figure S11E (Supporting Information) shows the total interaction energy in the system. With polyguanidine aggregates in the system, the interaction energy between the molecules also increased. The electrostatic interaction between polyguanidines is significantly stronger than van der Waals interactions (the Coulomb potential represents electrostatic interactions, with hydrogen bonding as the main type, whereas the Lennard‐Jones potential represents van der Waals interactions), which suggests that the stronger electrostatic interaction energy between the two molecules drives their self‐assembly in aqueous solvents to form stable nanoclusters (Figure S11F, Supporting Information). Finally, transmission electron microscopy (TEM; Figure 2D) revealed that each of PEG‐***b***‐P(Gu)1‐5, PLGA‐***b***‐P(Gu)1‐5, and PLA‐***b***‐P(Gu)1‐5 self‐assembled into nanoparticles. The sizes of PEG‐***b***‐P(Gu) and PLA‐***b***‐P(Gu) were approximately in the range of 150–200 nm, whereas those of PLGA‐***b***‐P(Gu) were 30–50 nm. These differences in particle size may be attributed to the ester bonds of lactic acid and glycolic acid in PLGA molecular chains, which endow these nanoparticles with stronger hydrophobic interactions and self‐assembly capabilities during nanoparticle formation. This leads to a tendency to form tighter particle structures and consequently smaller sizes. In addition, we used Dynamic Light Scattering (DLS) and PDI characterizations that evaluate the particle size distribution and aggregation behavior of these nanoparticles in aqueous solutions, to assess their stability. Based on these results (Figure S12, Supporting Information), we observe that the particle sizes of the nanoparticles remain relatively unchanged within the 7‐day period, exhibiting preferable colloidal stability.

### 2.3 Small Molecule Guanidines and PGNIs Ameliorate Lipid Accumulation In Vitro

In a cytotoxic test using the CCK8 assay, we found that the viability of HepG2 and MIHA cells treated with PGNIs was also facilitated, and kupffer cells is maintained at normal levels (Figure 2E; Figures S13A and S14, Supporting Information). To evaluate the effect of PGNIs on lipid deposition, the levels of triglycerides (TG) (Figure 2F; Figure S13B, Supporting Information) and Bodipy‐labeled lipid droplets (Figure 2G) were detected in HepG2 cells, and Oil Red O staining was performed (Figure 2G). All results indicated that both small‐molecule guanidine, represented by metformin, and large‐molecule guanidine PGNIs ameliorated liver lipid deposition in vitro, with PGNIs exhibiting a slightly better effect than small‐molecule guanidine.

### 2.4 PGNIs Ameliorate Liver Lipid Accumulation In Vivo

**Figure 3:**
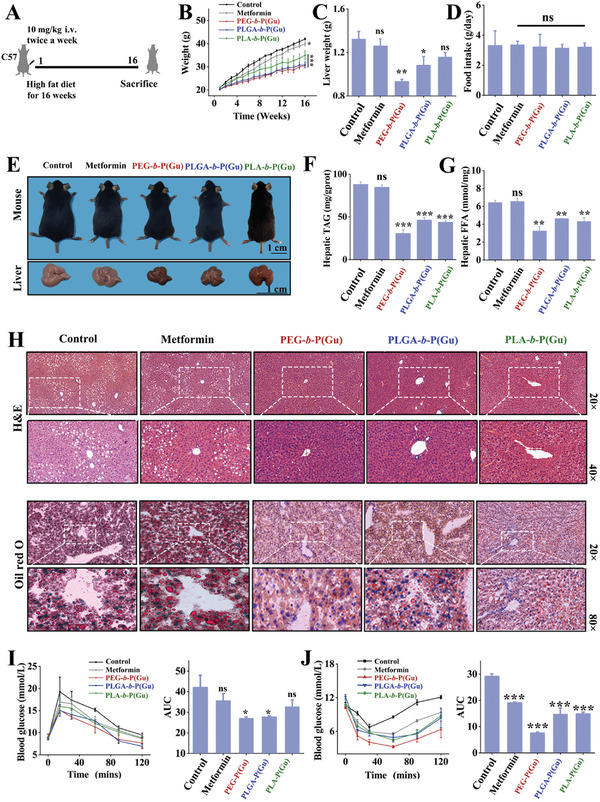
Amelioration of HFD‐induced lipid deposition in mice by polyguanidine nanomedicine administration. Small molecule guanidines (e.g., metformin) do not show this lipid lowering effect. A) Schematic of hepatic lipid deposition reduction in C57BL/6J mice and the treatment schedule when using PEG‐***b***‐P(Gu), PLGA‐***b***‐P(Gu), or PLA‐***b***‐P(Gu). B) Body weight, C) liver weight, and D) feed intake of C57BL/6 mice treated with different medicines. Bars represent the mean ± SD (n = 6). E) Images of the whole‐body and liver of mice at the end of treatment. Scale bar: 1 cm. F) Hepatic total triglyceride and G) free fatty acids levels in mice after 16 weeks of treatment with metformin, PEG‐***b***‐P(Gu), PLGA‐***b***‐P(Gu), or PLA‐***b***‐P(Gu). Bars represent the mean ± SD (n = 6). H) H&E and Oil Red O staining images of liver cells collected from mice administrated with metformin, PEG‐***b***‐P(Gu), PLGA‐***b***‐P(Gu), or PLA‐***b***‐P(Gu). I) Results of glucose tolerance tests (GTTs) and(J) insulin tolerance tests (ITTs) conducted on HFD‐fed mice before and after the administration of metformin and polyguanidine nanomedicine (n = 6). ^***^ *P* < 0.001; ^**^ *P *< 0.01; ^*^ *P* < 0.05. The P‐values are calculated using one‐way ANOVA.

Based on the above results, for subsequent experiments, we selected those numbered three for each type of PGNI. Because these PGNIs demonstrated a relatively prominent and consistent performance in ameliorating hepatic triglyceride levels. While PGNIs numbered 4 and 5 also showed some improvement in lipid deposition, this enhancement did not follow a pronounced dose‐response trend. Consequently, the selection of PGNI number 3 aligns more closely with the principles of economy and high efficiency. The capacity of small‐molecule guanidine and PGNIs to ameliorate lipid deposition was first investigated in the liver. As a proof of concept, HFD‐fed mice were intravenously (i.v.) injected with metformin and different PGNIs twice a week for 16 weeks (**Figure** **3**A). As Figure 3B,C show, the administration of PGNIs flattened the steep body and liver weight gains. This anti‐obesity effect was not caused by reduced food intake (Figure 3D); it was exclusively accounted for by the inhibition of lipid deposition in the body, liver, and serum by PGNIs, especially PEG‐***b***‐P(Gu) (Figure 3E–H; Figure S15, Supporting Information). On the contrary, at this dose, metformin did not have any effect. Moreover, PGNIs relieved insulin resistance (Figure 3I,J). To exclude the liver lipid‐lowering effects caused by the charge, other compositions, and sizes of the nanoparticles, a classic cationic dendritic polymer (P‐G3)^[^ ^26^ ^]^ and PEG were selected for administration to HFD‐fed mice. As depicted in Figure S16A–D (Supporting Information), PEG did not have any effect on body and liver weights, while suppression was observed in mice that were administered P‐G3; however, it was not as significant as in the mice treated with PGNIs. In terms of improving lipid deposition in the liver and serum, there was no significant difference between the P‐G3 and PEG‐administered groups compared to the HFD group (Figure S16E–H, Supporting Information). Based on these results, we speculated that the guanidine group in PGNIs might induce this pharmacological activity.

### 2.5 Biosafety of PGNIs

In addition to studying the ameliorating effect of PGNIs on lipid deposition, their safety was assessed in animal toxicology studies (Figure S17, Supporting Information). No abnormal levels of aminotransferase (ALT), aspartate aminotransferase (AST), urea nitrogen (BUN), or creatinine (S‐Cr) were observed in mice that were administered PEG‐***b***‐P(Gu), PLGA‐***b***‐P(Gu), or PLA‐***b***‐P(Gu). Hematoxylin and eosin (H&E) staining of major organ sections also indicated no observable influence on other organs.

### 2.6 Biodistribution In Vitro and In Vivo

**Figure 4:**
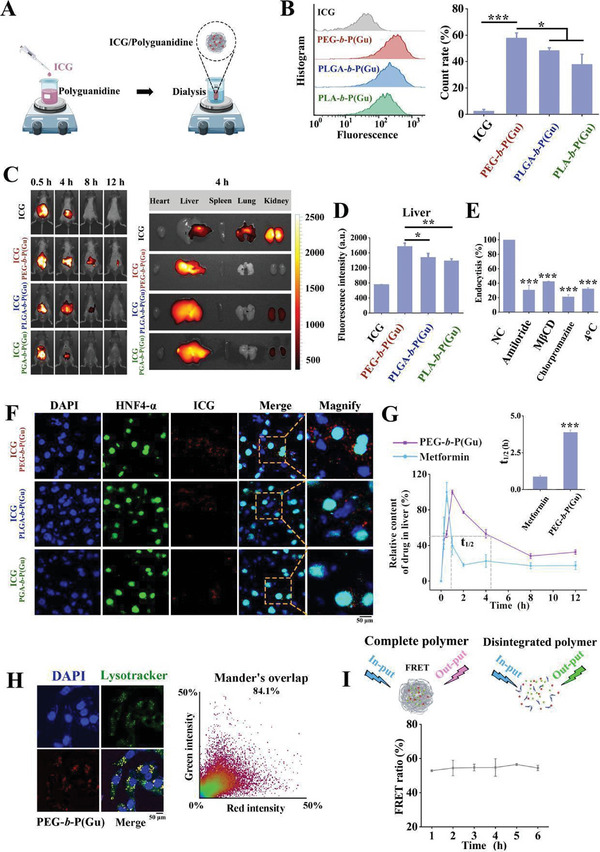
Biodistribution and pharmacokinetics profile of polyguanidine nanomedicine in vitro and in vivo. A) Schematic of self‐assembly of polyguanidine nanomedicine with the corresponding fluorescent labels. B) FCM analysis and quantification of polyguanidine nanomedicine cellular uptake in HepG2 cells (n = 3). C) Luminescence images D) quantification of C57BL/6J mice following treatment with PEG‐***b***‐P(Gu)/ICG, PLGA‐***b***‐P(Gu)/ICG, or PLA‐***b***‐P(Gu)/ICG in vitro and in vivo (n = 3) E) Inhibition of endocytosis by different inhibitors, including amiloride, mβCD, chlorpromazine, and 4 °C for detecting cellular uptake pathways (n = 3). F) Immunofluorescence image of mouse liver treated with PEG‐***b***‐P(Gu)/ICG (blue: nucleus; green: hepatic parenchymal cell; red: PEG‐***b***‐P(Gu); scale bar: 50 µm). G) Drug concentration‐time curve of PEG‐***b***‐P(Gu) and metformin in mouse liver. Bars represent the mean ± SD (n = 6). H) CLSM and Mander's overlap of PEG‐***b***‐P(Gu) cellular uptake and accumulation in the lysosome. Blue: nucleus; green: lysosome; red: PEG‐***b***‐P(Gu); yellow: merged. Scale bars: 50 µm. I) FRET ratio of particles prepared with Dio and Dil after incubation in HepG2 cells. Bars represent the mean ± SD (n = 3). ^***^ *P* < 0.001; ^**^ *P* < 0.01; ^*^ *P* < 0.05. The P‐values are calculated using one‐way ANOVA.

To explore the reasons for the inconsistent lipid‐lowering effects of guanidines with different molecular weights or prepolymer types in vitro and in vivo, we investigated the dynamic state of the PGNIs in vivo. First, PGNIs labeled with indocyanine green (ICG/PEG‐***b***‐P(Gu), ICG/PLGA‐***b***‐P(Gu), and ICG/PLA‐***b***‐P(Gu)) were produced (**Figure** **4**A). The following experiment showed that the three different aggregation types of PGNIs exhibited different uptake abilities in liver cells (Figure 4B). Four groups of animals were given a single dose of ICG: ICG/PEG‐***b***‐P(Gu), ICG/PLGA‐***b***‐P(Gu), or ICG/PLA‐***b***‐P(Gu). As shown in Figure 4C, the three types of PGNIs showed significant liver accumulation at 0.5–4 h. 8 h after injection, fluorescence in the liver gradually decreased. In contrast, the ICG group exhibited rapid and complete metabolism, because fluorescence was observed in the kidneys after 4 h. In addition, the ex vivo fluorescence image and quantitative diagram (Figure 4D) of the excised organs further confirmed that ICG/PEG‐***b***‐P(Gu) showed a higher fluorescence signal in the liver than in the other groups. Next, immunofluorescence staining was performed on the liver at 4 h after dose administration to further explore the dynamics of PGNIs within the liver. The PEG‐***b***‐P(Gu)/ICG group showed a higher localization in hepatocytes (marked as HNF4‐α) compared to other groups (Figure 4F), which might explain why PEG‐***b***‐P(Gu) exhibits better lipid‐lowering effects in vivo. Hence, PEG‐***b***‐P(Gu)3 was used for further experiments. As shown in Figure 4E, three endocytosis inhibitors, amiloride, methyl‐β‐cyclodextrin (MβCD), and chlorpromazine, were employed to verify the macropinocytosis, caveolae‐mediated endocytosis, and clathrin‐mediated endocytosis efficiency, respectively.^[^ ^27^ ^]^ The endocytosis efficiency was significantly decreased in cells treated with chlorpromazine, suggesting that PEG‐***b***‐P(Gu) entered the cells via macropinocytosis and caveolae‐ and macropinocytosis‐related pathways, as all inhibitors slowed down cellular uptake. An analysis of the drug concentration‐time curve suggested that the half‐life of PEG‐***b***‐P(Gu) was ≈4 h longer than that of metformin (0.5 h) (Figure 4G). At 4 h, PEG‐***b***‐(Gu) was mainly localized in the lysosomes within the liver cells (Figure 4H) rather than in other organelles (Figure S18, Supporting Information). Finally, fluorescence resonance energy transfer (FRET) was used to investigate the action of nanoparticles within cells. This process occurs when the emission spectrum of the donor overlaps with the absorption spectrum of the acceptor and the donor and acceptor are in close proximity.^[^ ^28^ ^]^ As shown in Figure 4I, the ratio of the FRET fluorescence pair did not undergo any significant change during the time in which the nanoparticles resided within the lysosomes, suggesting that the nanoparticles produced the effect as intact particles.

### 2.7 PEG‐*b*‐P(Gu) Inhibits Lysosomal Activity and Raises Lysosomal pH

**Figure 5:**
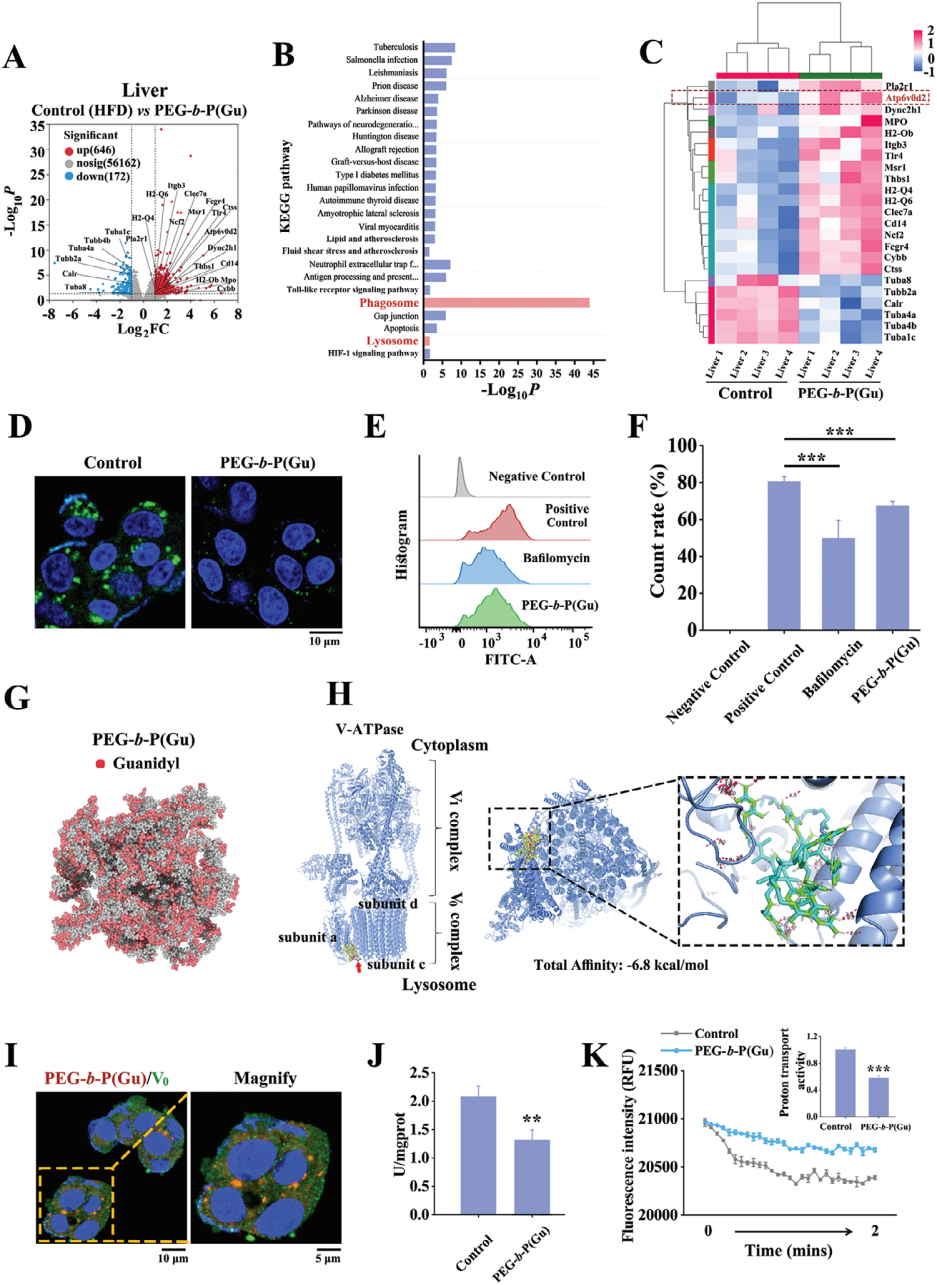
Inhibition of lysosomal V‐ATPase activity by PEG‐***b***‐P(Gu), which targets the V_0_ region. A) Volcano plots of differentially expressed genes in HFD‐fed mouse liver and comparison with those of PEG‐administered mouse liver. FC, fold change. The upregulated and downregulated genes are shown in red and blue, respectively. B) KEGG pathway enrichment analysis and the corresponding scores. The enrichment score representing the enrichment degree among the differential pathways is shown in ‐log10. C) Heat map showing the expression levels of 23 phagosome relative genes in mouse liver after treatment with PEG‐***b***‐P(Gu). The control group consists of mice fed with HFD. D) CLSM image of the deacidification property of lysosomes in HepG2. The control group means palmitic acid loading of HepG2 cells; fluorescence intensities of left: lysosensor; right: PEG‐***b***‐P(Gu); scale bar: 10 µm. E) FCM analysis and F) quantification of the deacidification property of lysosomes in HepG2 after treatment with V‐ATPase inhibitor bafilomycin and PEG‐***b***‐P(Gu) (n = 3). The positive control group means palmitic acid loading of HepG2 cells. G) Assembly structure of PEG‐***b***‐P(Gu) after molecular dynamics simulation (red represents guanidine, gray represents the other groups). H) Molecular docking between PEG‐***b***‐P(Gu) and V‐ATPase (green stripes represent the guanidine groups and red dashed lines represent the interaction forces). I) Localization of PEG‐***b***‐P(Gu) and V_0_ region in HepG2 (red: PEG‐***b***‐P(Gu); blue: nucleus; green: V_0_ region of V‐ATPase; yellow: merged; scale bar: 10 µm; and magnification: 5 µm). J) The vitality of V‐ATPase and K) proton transport activity of HepG2 after treatment with PEG‐***b***‐P(Gu) (n = 3). The control group means palmitic acid loading of HepG2 cells. ^***^ *P* < 0.001; ^**^ *P* < 0.01; ^*^ *P* < 0.05; statistical analysis was performed using one‐way ANOVA.

In addition to the dynamic state in vivo, the mechanisms underlying the pharmacological effects of polyguanidine must be investigated. The transcriptome differences in liver tissues were analyzed using RNA sequencing. The volcano plot (**Figure** **5**A) revealed that, compared with the HFD‐fed group (control), 646 genes were upregulated, and 172 genes were downregulated after the administration of PEG‐***b***‐P(Gu). KEGG analysis indicated that phagocytosis‐related pathways were significantly activated (Figure 5B), and this activation was not attributed to inflammation (Figure S19, Supporting Information). Among these phagocytosis‐related proteins, the increase in the transcriptome (Figure 5C) and protein expression (Figure S20, Supporting Information) of ATP6V0D2, a “d” subunit of V‐ATPase, has attracted our attention. V‐ATPase primarily controls the acidic environment of lysosomes and is closely associated with their activity.^[^ ^29^ ^]^ In its V_0_ domain, V‐ATPase is mainly divided into multiple subunits such as “a”, “c”, and “d”. The “a” subunit is the core component of V‐ATPase and directly participates in the conduction of H^+^. The “c” subunit is a major component of the proton channel, while the “d” subunit is mainly used to maintain the stability of the proton channel,^[^ ^30^, ^31^ ^]^ furthermore, study have also shown evidence that ATP6V0D2 can promote the fusion of autophagosomes and lysosomes.^[^ ^32^ ^]^ Therefore, despite the lysosomal pathway not being prominent in the KEGG analysis (Figure 5C), as part of the phagocytic pathway, it and V‐ATPase may still play an important role. Thus, we further examined lysosomal activity in HepG2 cells after treatment with PEG‐***b***‐P(Gu) and observed a significant downregulation (Figure 5D–F) and pH increase (Figure S21, Supporting Information), but the pH remained within the optimum range of lysosome (4.5–5.0). An increase in lysosomal pH is generally detrimental to lipid degradation and autophagy,^[^ ^33^ ^]^ current research even developed nanoparticles that directly act on lysosomal acidification.^[^ ^34^ ^]^ However, the effect of V‐ATPase inhibition on lipid deposition is not solely dependent on lysosomal pH modulation. Rather, it may be attributed to V‐ATPase's intricate roles in cellular signaling and lipid metabolism regulation. But what is even more important is that, since ATP6V0D2 does not participate in proton transport within the V‐ATPase, it is worthwhile to further investigate exactly how PEG‐***b***‐P(Gu) leads to an increase in the lysosomal pH value, which subsequently upregulates ATP6V0D2, there may be a feedback regulation mechanism involved.

### 2.8 PEG‐*b*‐P(Gu) Targets V_0_ Region, Inhibits ATPV0A, and Triggers ATPV0D2 Upregulation

Therefore, we docked the PEG‐***b***‐P(Gu) molecule with V‐ATPase to determine the approximate range of interaction sites. As shown in Figure 5G,H, guanidine groups are distributed throughout all the polymers and interact with the V_0_ region of V‐ATPase (the V_0_ region exists in the inner leaflets of the lysosome, while V_1_ is located in the outer leaflets^[^ ^29^ ^]^). Further fluorescence imaging (Figure 5I) also revealed an interaction between PEG‐***b***‐P(Gu) and the V_0_ domain. Then, we used ATP6V0A1, ATP6V0C, and ATP6V0D2 recombinant proteins to dock with PEG‐***b***‐P(Gu) through surface plasmon resonance (SPR). As anticipated by the molecular dynamics simulations, the SPR results (Figure S22, Supporting Information) showed that although all three proteins could bind to PEG‐***b***‐P(Gu) to varying degrees, but in terms of dissociation constants (K_D_), ATP6V0A1 had a higher affinity than ATP6V0C, which in turn had a higher affinity than ATP6V0D2. This suggests that PEG‐***b***‐P(Gu) is likely to target the “a” subunit of V‐ATPase. In addition, the docking of PEG‐***b***‐P(Gu) with V‐ATPase also resulted in a decrease in V‐ATPase activity (Figure 5J) and H^+^ pumping capability (Figure 5K), as the “a” subunit is the core component of V‐ATPase and directly participates in the conduction of H^+^.^[^ ^31^ ^]^ Due to the observed weakening of the H^+^ pumping ability of V‐ATPase, we speculate that PEG‐***b***‐P(Gu) may inhibit the H^+^ conduction of the “a” subunit based on its function. After sensing this inhibition, the cell acts on the ATPV0D2 subunit through feedback regulation to maintain the stability of the proton channel, which may be the reason why we observed high expression of ATPV0D2 at both the transcriptomic and protein levels.

### 2.9 PEG‐*b*‐P(Gu) Increases the Energy Expenditure by Activating AMPK Phosphorylation

**Figure 6:**
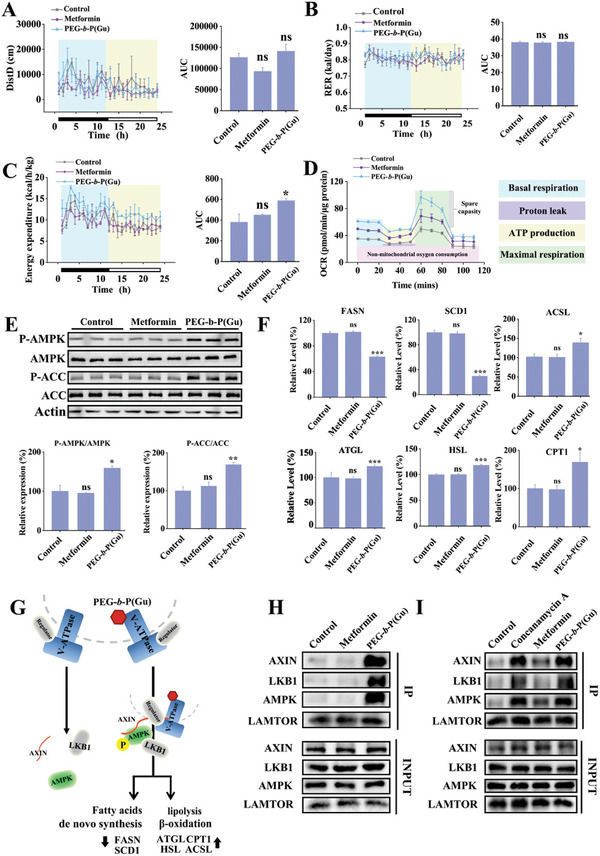
PEG‐***b***‐P(Gu) increases the energy expenditure in vitro and in vivo by activating AMPK. A) Activity level, B) respiratory quotient, and C) energy consumption of C57BL/C mice after receiving 10 mg k^−1^g PEG‐***b***‐P(Gu) i.v. twice a week since the beginning of HFD feeding. The mice were singly housed in metabolic cages for calorimetric analysis (n = 6). D) Basal respiration, maximal respiration, and ATP production were calculated through oxygen consumption rate (OCR) in HepG2 cells as measured using a Seahorse XF24 analyzer (n = 3). () Expression and phosphorylation levels of AMPK and ACC protein, and their quantitative data in mouse liver (n = 3). F) qPCR analysis of the gene expression of fatty acid synthesis, lipolysis, and β‐oxidation markers in mouse liver (n = 6). G) Schematic diagram showing that PEG‐***b***‐P(Gu) binds to V‐ATPase via interacting with ATPase V_0_ region, thereby inhibiting the activity of V‐ATPase and then activating AMPK pathway. H) Interaction among LAMTOR1, AXIN, LKB1, and AMPK detected by CO‐IP in mouse liver and I) in HepG2 cells. ^***^ *P* < 0.001; ^**^ *P* < 0.01; ^*^ *P* < 0.05; statistical analysis was performed using one‐way ANOVA.

Subsequently, we explored how the inhibition of V‐ATPase affects energy metabolism both in vivo and in vitro. An indirect calorimetric analysis of energy homoeostasis revealed that PEG‐***b***‐P(Gu) treatment increased energy expenditure without affecting locomotor activity or the respiratory exchange ratio in mice (**Figure** **6**A–C). Furthermore, we measured the oxygen consumption rate in HepG2 cells and found that PEG‐***b***‐P(Gu) significantly increased basal respiration, maximal respiration, and adenosine triphosphate (ATP) production compared to the control and metformin groups, suggesting that PEG‐***b***‐P(Gu) treatment increased energy metabolism and fatty acid oxidation, thereby contributing to reduced lipid accumulation in the liver. (Figure 6D; Figure S23, Supporting Information). Based on these results, we focused on AMPK, as it is a key metabolic regulator that plays a pivotal role in maintaining cellular energy homeostasis.^[^ ^35^ ^]^ Once activated (phosphorylation level increases), AMPK stimulates catabolic pathways that generate ATP while inhibiting anabolic pathways that consume ATP.^[^ ^36^ ^]^ In addition, activated AMPK inhibits the activity of acetyl‐CoA carboxylase (ACC) by increasing its phosphorylation.^[^ ^10^ ^]^ The suppression of the AMPK pathway can exacerbate fatty liver changes and lipid metabolic disorders, thereby playing a significant role in the development and progression of NAFLD.^[^ ^37^ ^]^ According to Figure 6E, the phosphorylation of AMPK or ACC increased in mouse livers following PEG‐***b***‐P(Gu) treatment, suggesting that AMPK was activated, and ACC was inhibited. Consistently, the expression of de novo fatty acid synthesis markers in the liver was suppressed, including FASN and SCD1,^[^ ^38^ ^]^ whereas lipolysis and fatty acid oxidation markers (ACSL, ATGL, HSL, and CPT1)^[^ ^39^ ^]^ were dramatically increased (Figure 6F). Notably, AMPK activation was also caused by the interaction of the inhibited V‐ATPase with LKB1/AXIN and AMPK in the mouse livers after PEG‐***b***‐P(Gu) treatment (Figure 6G,H). Similarly, after the treatment of V‐ATPase antagonists (concanamycin A),^[^ ^40^ ^]^ the interaction between these proteins ceased on HepG2 cells (Figure 6I). The AMPK inhibitors (compound C)^[^ ^41^ ^]^ also inhibited the effects of PEG‐***b***‐P(Gu) on HepG2 cells (Figure S24A–C, Supporting Information).

## 3 Conclusion

In the realm of innovative drug development, traditional drugs may face challenges amidst the rapid technological advancements and emergence of novel therapies. However, it is also essential to recognize that even compounds based on known mechanisms, through meticulous structural optimization and targeted design, may uncover novel biological effects or enhance efficacy, thereby finding new application spaces in therapeutic areas. In this study, through the synthesis and screening of a series of PGNIs, we identified that polyguanidine can greatly ameliorate lipid accumulation compared to small‐molecule guanidine. In addition to lipid lowering, the pharmacokinetics and interaction sites of guanidines are shifted because of nanoization resulting from polymerization. PGNIs are mainly located in hepatocytes and are taken up by lysosomes. Inside lysosomes, PGNI combined with the “a” subunit in V_0_ residue of V‐ATPase inhibits its activity and feedback the increase of ATP6V0D2. Subsequently, V‐ATPase and Regulator (LAMTOR) allow LKB1 and AXIN to be recruited to activate AMPK. This process further decreases de novo fatty acid synthesis, promotes lipolysis and fatty acid oxidation, and ameliorates liver lipid accumulation. Although this mechanism similarities with metformin in certain aspects, we aspire to uncover its potential in reducing lipid accumulation through further investigation, aiming to provide more diverse and effective treatment options for NAFLD through a multi‐faceted approach. In summary, we discovered a novel pharmacological interaction mode between guanidine‐based compounds and potential target sites for fatty liver disease, which may be applicable to a wider range of high‐solubility and low‐permeability drugs, thereby yielding better efficacy in treating diseases.

## Conflict of Interest

The authors declare no conflict of interest.

## Supporting information



Supporting Information

## Data Availability

The data that support the findings of this study are available from the corresponding author upon reasonable request.
