# Supplementary material for: Guanidine‐Derived Polymeric Nanoinhibitors Target the Lysosomal V‐ATPase and Activate AMPK Pathway to Ameliorate Liver Lipid Accumulation
Source: Adv Sci (Weinh). 2024 Nov 5;12(1):2408906. doi: 10.1002/advs.202408906 (PMC11714212; doi:10.1002/advs.202408906)
Supplement: Supplementary file 1 — Supporting Information [file ADVS-12-2408906-s001.docx]

**Guanidine-Derived Polymeric Nanoinhibitors Target the Lysosomal V-ATPase and Activate AMPK Pathway to Ameliorate Liver Lipid Accumulation**

Yunfei Zhao^#^, Ke Hu^#^, Fangliang Wang^#^, Lulu Zhao^#^, Yu Su, Jun Chen, Gang Zou，Liming Yang, Li Wei, Mengjiao Deng, Yunyu He, Ping Wang, Xiong Z Ruan*, Yaxi Chen*, Chao Yu*

Y. F. Zhao, F.L. Wang, L.L. Zhao, J. Chen, G. Zou, L.M. Yang, M.J. Deng, Y.Y. He, C. Yu

Chongqing Medical University, College of Pharmacy, Chongqing Key Laboratory for Pharmaceutical Metabolism Research, Chongqing pharmacodynamic evaluation engineering technology research center, Chongqing 400016, P.R. China

K. Hu, Y. Su, P. Wang, L. Wei, X.Z Ruan, Y.X Chen

Centre for Lipid Research & Chongqing Key Laboratory of Metabolism on Lipid and Glucose, Key Laboratory of Molecular Biology for Infectious Diseases (Ministry of Education), Institute for Viral Hepatitis, Department of Infectious Diseases, the Second Affiliated Hospital, Chongqing Medical University, 400016, Chongqing, China

# Yunfei Zhao, Ke Hu, Fangliang Wang and Lulu Zhao contributed equally to this work.

*Corresponding authors: Xiong Z Ruan, Yaxi Chen and Chao Yu

E-mail: x.ruan@ucl.ac.uk; chenyaxi@cqmu.edu.cn; yuchao@cqmu.edu.cn

**Experimental Section**

**Materials**

Methacrylic acid, N-hydroxysuccinimide ester, amiloride hydrochloride, and chlorpromazine were purchased from Aladdin (China). Bis(thiobenzoyl) disulfide was purchased from TCI (Tokyo, Japan). 4,4′-azobis (4-cyanovaleric acid), dicyandiamide, EDTA (E6758), HEPES (H4034), ethylenediamine, 2,2'-azobis-(2-methylpropionitrile) (AIBN), 1-(3-dimethylaminopropyl)-3-ethylcarbodiimide (EDC), and N-hydroxy succinimide (NHS) were purchased from Sigma-Aldrich (USA). MeO-PEG-NH2, PLGA-NH2, and PLA-NH2 were purchased from Xian Ruixi Bio (Beijing, China). Indocyanine green (ICG) was purchased from Yuanye (China). Methyl-β-cyclodextrin was purchased from TargetMol (USA). A rodent diet containing 60 Kcal% fat (D12492) was obtained from Research Diets. Phospho-AMPKα (2535S), LAMTOR1 (8975S), and Axin1 (2087S) were obtained from CST. Bodipy (D3821) and lysosome sensor green (A66436) were purchased from Thermo Fisher Scientific. LKB1 (10746-1-AP), AMPK (10929-2-AP), ACTIN (81115-1-RR), HRP-conjugated Affinipure goat anti-rabbit IgG (H+L) (SA00001-2), and a hydrophobic IHC pen (PR30013) were supplied by Proteintech. Human ATP6V0A1 (H00000535-P01) and ATP6V0C (H00000527-P01) Recombinant Protein were purchased from Abnova Biotechnology. Human ATP6V0D2 (523519) was obtained from NovoPro. Hoechst 33342 (C1028), ER-tracker green (C1042S), lyso-tracker green (C1047S), mito-tracker (C1048) and ProLong Diamond (DAPI) (P0131) were purchased from Beyotime Biotechnology. KCl (P433488), glycerol (G116209), MgCl_2_ (M434102), NADH (N612137), concanamycin A (C102380), KCl (P433501), and KOH (P301749) were purchased from Aladdin. Glucose (D769078), NaOH (S835850), phosphoenolpyruvate (P796251), pyruvate kinase (P915244), lactate dehydrogenase (L915272), FITC-dextran (F861785), metformin (M794099), [1,1'-dioctadecyl-3,3,3',3'-tetramethylindocarbocyanine perchlorate](https://www.chemsrc.com/en/cas/41085-99-8_751013.html) (Dil), and [3,3'-dioctadecyloxacarbocyanine perchlorate](https://www.chemsrc.com/en/cas/34215-57-1_26946.html) (Dio) were supplied by Macklin. Insulin from bovine pancreas (11070-73-8), NaCl (S8210), Oil Red O staining kit (G1261-2, G1262-4), lysosome isolation kit (EX2670), DMSO (D8371), and BSA (A8020) were purchased from Solarbio. The triglyceride assay kit (A110-1-1) and non-esterified free fatty acid assay kit (A042-2-1) were obtained from Nanjing Jiancheng Bioengineering Institute. The PrimeScript RT Master Mix (RR036A) was purchased from Takawa. The SYBRPRIME qPCR Kit (BG0014) was obtained from Bioground. P-ACC (AF3421) and ACC (AF6421) were supplied by Affinity. ATPV0D2 (bs-12548R) was purchased from Bioss (USA). The CCK-8 kit (K1018) was obtained from APExBIO. Mg-ATP (AA0271) was obtained from G-Clone. Dorsomorphin (HY-13418A) was purchased from MedChem Express. Dynabead protein A/G (B23202) and bafilomycin A1 (S1413) were purchased from Selleck Chemicals.

**Synthesis of PEG-CPADN, PLGA-CPADN, PLA-CPADN**

PEG-NH_2_ (50 μmol, Mw: 5000), PLGA-NH_2_ (50 μmol, Mw: 5000), PLA-NH_2_ (50 μmol, Mw: 5000), and NHS-activated CPADN (1 mmol) were dissolved in dichloromethane and stirred for 12 h at room temperature (r.t.). The obtained mPEG-CPADN was recrystallized in cold diethyl ether three times to obtain a pink substance.

**Synthesis of PEG-*b*-P(Gu)1-5, PLGA-*b*-P(Gu)1-5, PLA-*b*-P(Gu)1-5**

Different proportions of PEG-CPADN, PLGA-CPADN, PLA-CPADN, N-(2-(3-carbamimidoylguanidino)ethyl)methacrylamide (GuC), and AIBN (5 mmol) were dissolved into DMSO and sealed under nitrogen. Then, the reaction is proceeded at 70 °C for 1 day. Next, the reaction mixture was dialyzed in water for 1 d. The product was obtained after freeze-drying. These proportions are shown in Fig. S2.

**MD docking and simulation**

For MD docking, Autodocks 1.5.7 was used to predict the interaction between PEG-***b***-P(Gu) and V-ATPase. MD simulations were performed using GROMACS 2019.6. During the MD simulation, all hydrogen bonds involved were constrained using the LINCS algorithm with an integration step size of 2 fs. The particle-mesh Ewald (PME) method was used to calculate electrostatic interactions. The cutoff value for non-bonded interactions was set at 10 Å and updated every 10 steps. Initially, the steepest descent method was applied to minimize the energy of the system and eliminate excessively close contacts between the atoms. Subsequently, 100 ps of NVT and NPT equilibrium simulations were performed at 298.15 K. Finally, a 100 ns MD simulation was conducted on the system, with configurations saved every 25 ps. The simulation results were visualized using the embedded programs Gromacs and VMD.

**Preparation of PGNIs**

PEG-***b***-P(Gu)1-5, PLGA-***b***-P(Gu)1-5, and PLA-***b***-P(Gu)1-5 in solid form were dissolved in deionized water for self-assembly into PGNIs. For ICG- or Dio/Dil-labeled PGNIs, the predetermined PGNIs were dissolved in 1 mL of deionized water. Then, 200 μL of fluorescent molecules (2 mg/mL ICG in DMSO or 0.5 mg/mL:1 mg/mL Dio:Dil) was dropped into the PGNIs solution under stirring at 300 rpm for 30 min. Next, the mixture was purified by dialysis against deionized water for 24 h to remove free molecules.

**Characterization of PGNIs**

The structures of the PGNIs were observed using a Bruker Avance III 600 MHz nuclear magnetic resonance (NMR) spectrometer (Bruker, Germany). The average molecular weight of the PGNIs was determined using LC20 gel permeation chromatography (GPC, Shimadzu, Japan), and their zeta potential was determined using DLS (NanoZS90, Malvern Instrument, UK). The morphology of the nanoparticles was observed using transmission electron microscopy (TEM; JEM1200EX, JEOL, Japan).

**Cell culture**

Human hepatocellular carcinoma HepG2 cells were cultured in DMEM supplemented with 10% FBS and 1% penicillin/streptomycin. The cells were cultured at 37 ℃ in a humidified atmosphere containing 5% CO_2_.

**Cell viability**

Cell viability was measured by incubating HepG2 cells at varying concentrations of PGNIs for 24 h. The Cell Counting Kit-8 was then used according to the manufacturer's instructions. A calibration plot was constructed using positive and negative controls.

**Isolation of mouse Kupffer cells**

Proceeding with the experimental procedure ^[1]^, the mouse liver was extensively exposed, and were perfused with a digestion solution containing collagenase IV. Subsequently, the liver was carefully isolated and transferred to a cell culture medium-filled petri dish. Tweezers were utilized to grasp and shake the liver, aiding in the release of cells. The resulting cell suspension was transferred to a 50 ml centrifuge tube and spun down at 20 rcf for 3 minutes. The supernatant was spun down again, and varying concentrations of percoll were employed to further purify target cells. Kupffer cells were identified by flow cytometry using Alexa Fluor® 647 anti-mouse Tim-4 Antibody.

**Cellular uptake study**

HepG2 cells were seeded in 24-well plates and cultured overnight in 500 μL of DMEM supplemented with 10% FBS. Then, the culture medium was replaced with 500 μL of 10% FBS DMEM, and the assembled PGNIs were added and mixed to give final concentrations of 10 μg/mL.

**Measurement of intracellular triglyceride**

The treated HepG2 cells were cultured in serum-free medium with 200 μM palmitic acid (PA) for 12 h. The cells were then washed three times; the intracellular lipids were extracted in isopropanol; and the TG levels were measured using an enzymatic assay.

**Confocal microscopy imaging**

DAPI was used to label the HepG2 nuclei; lyso-tracker, mito-tracker, and ER-tracker were used to label the corresponding organelles; and ICG and Dio/Dil were used to label PEG-***b***-P(Gu). For ATPV0, treated cells were stained with anti-ATPV0 and a secondary fluorescent antibody. The HepG2 cells were seeded into wells at a density of 50%-60% and transfected as described above. The cells were then washed three times and observed under a confocal microscope.

**Surface Plasmon Resonance (SPR)**

10× PBS-P+ was used as a running buffer for the SPR experiments. PEG-***b***-P(Gu) was prepared diluted from 2.5 to 0.07813 μM in the running buffer. Sample cycles consist of contact time 60 s, dissociation time 120 s, and regeneration pulse (50% DMSO) to remove bound analyte. All experiments were carried out at room temperature. Raw SPR data were fitted into the sensorgrams obtained at six different concentrations using the Biacore X100 Evaluation Software. Evaluation of the association and dissociation rates was performed by applying linear or nonlinear fitting algorithms to the binding data.

**High Performance Liquid Chromatography (HPLC)**

The chromatographic column was composed of diamonsil C18 (250 mm × 4.6 mm, 5 µm) and a mobile phase: 0.1% NaH_2_PO_4_-acetonitrile (10:90). The flow rate was 1.0 mL/min, and the detection wavelength was 236 nm.

C57BL/6J mice were administered metformin via intravenous injection. Then, liver homogenates were obtained at 0.25, 0.5, 1, 2, 4, 8, and 12 h for testing.

**Flow cytometry**

The treated cells were washed three times with PBS, trypsinized, and were then resuspended in PBS for flow cytometry analysis. To quantitatively assess lysosome pH, the cells were subjected to a designated Cellular pH Calibration buffer treatment, and the fluorescence intensity of Lysosensor was precisely measured using flow cytometry. Based on the obtained data, a standard curve of Lysosensor fluorescence ratio to pH was established.

**Western blotting**

Protein extracts from the liver tissue or cells were lysed in RIPA buffer, and 20 μg of the protein was used for western blotting. The following primary antibodies were used: anti-AMPK, anti-p-AMPK, anti-ACC, and anti-p-ACC.

**Co-immunoprecipitation**

Proteins were extracted from cells and tissues using RIPA. Then, LKB1, AXIN, and AMPK were immunoprecipitated using anti-LKB1, anti-AXIN, and anti-AMPK antibodies coupled with protein G magnetic beads. All immunoprecipitated samples were washed three times with lysis buffer. Then, the bound proteins were eluted by boiling for 5 min in SDS buffer. Rabbit IgG was used as the negative control. Immunoprecipitated proteins were further analyzed by western blotting.

**Real-time PCR**

Total RNA from the tissues and cells was extracted using the TRIzol reagent. RT-PCR was performed using the SYBR Green PCR Master Mix with the specific primer sets shown in Fig. S20. b-actin was used as the internal reference gene, and the relative expression of the genes was analyzed using the 2-DD Ct method.

**Animals**

C57BL/6J mice were provided by the Experimental Animal Center of Chongqing Medical University (ethical approval statement 2021015). All animal procedures were performed in accordance with the National Institute of Health’s Guidelines for the Care and Use of Laboratory Animals. The mice were kept under a constant 12 h light-dark cycle with unrestricted access to food and water and received either NCD or HFD containing 60 kcal% fat for 16 weeks (n = 6).

**Biodistribution *in vivo***

Free ICG (100 μL) or PEG-***b***-P(Gu)/ICG were intravenously administered to the mice. Imaging was performed at 0.5, 4, 8, and 12 h after the I.V. injection and tracked using an *in vivo* imaging system. After live imaging, the mice were sacrificed, and their major organs, including the liver, lung, spleen, kidney, and heart, were dissected from the mice, followed by the determination of fluorescence intensity.

**RNA-seq**

PBS- or PEG-***b***-P(Gu)-fed C57BL/6J mice were sacrificed after feeding with HFD for 16 weeks, and their livers were collected for high-throughput transcriptome sequencing conducted by Majorbio BioTech Co., Ltd.

**GTT and ITT**

The mice were subjected to a glucose tolerance test (GTT) after 16 h of fasting and received an intravenous injection of a glucose solution (1.5 g/kg). Blood was collected from the tail, and the glucose levels of the mice were measured at 0, 15, 30, 60, and 120 min after injection. For the insulin tolerance test (ITT), the mice underwent fasting for 12 h and were then injected with insulin (0.75 U/kg).

**Oil Red O staining**

The cells or livers were collected after treatment with PBS, metformin, PEG-***b***-P(Gu), PLGA-***b***-P(Gu), or PLA-***b***-P(Gu) after washing and permeabilization with 4% paraformaldehyde for 20 min. Oil Red O staining was performed. Image analysis was performed using Image-Pro Plus 6.0.

**Histological Analysis**

The C57BL/6J mice, after being injected with PBS, metformin, PEG-***b***-P(Gu), PLGA-***b***-P(Gu), PLA-***b***-P(Gu), P-G3, or PEG, were sacrificed, and their major organs, including the heart, liver, spleen, lungs, and kidneys, were extracted. The tissues were fixed in 10% formalin and embedded in paraffin. Sections of the tissues were stained with hematoxylin and eosin or Oil Red O.

**Tests of Biochemical Parameters**

The serum and liver levels of TG, TC, ALT, AST, BUN, and S-Cr were determined using commercial kits (refer to the Materials section for details) according to the manufacturer's instructions.

**Lysosomal activity analysis**

Lysosomes from HepG2 cells collected in three 10-cm dishes were purified using a lysosome isolation kit according to the manufacturer’s instructions. They were then suspended in an assay buffer (50 mM NaCl, 30 mM KCl, 20 mM HEPES-NaOH, pH 7.0, 10% (v/v) glycerol, 1 mM MgCl_2_, 1.5 mM phosphoenolpyruvate, 0.35 mM NADH, 20 U/mL pyruvate kinase, and 10 U/ml lactate dehydrogenase) with 5 μM concanamycin A or DMSO and warmed at 37 °C for 10 min. The assay was initiated by the addition of 5 mM ATP, and OD_341_ was continuously recorded.

**V-ATPase proton transport activity**

HepG2 cells were incubated in DMEM supplemented with 2 mg/mL FITC-dextran on ice for 5 min and then transferred to a 37 °C for 30 min. The cells were washed three times with warm DMEM and incubated for 30 min at 37 °C to allow the transfer of FITC-dextran to the lysosomes. The liver cells of the mice were collected, and the lysosomes were purified as described above. These lysosomes were resuspended in the assay buffer (20 mM HEPES, 1 mM EDTA, 125 mM KCl, pH 7.5 with KOH) and then incubated for 1 h on ice. Next, 5 μM concanamycin A or DMSO was added, and the mixture warmed at 37 °C for 10 min. Fluorescence was recorded at excitation and emission wavelengths of 490 and 520 nm, respectively. The initial slope of the fluorescence curve was measured after the addition of 5 mM Mg-ATP.

**Energy metabolism analysis**

The experimental mice were housed for 2 weeks in TSE PhenoMaster metabolic cages for acclimatization to the new cage environment. Their food and water intake, movement distance, respiratory exchange ratio (RER), and energy expenditure were measured throughout the 48 h metabolic profiling studies. Food and water were provided, and all the mice were housed under a 12 h light-dark cycle.

The oxygen consumption of the treated HepG2 cells was measured on a Seahorse XF24 analyzer at 37 °C. The cells were seeded on a Seahorse XF24 analyzer plate. After 4 h, the medium was replaced with the Seahorse assay medium. The oxygen consumption rate (OCR) of the cells was measured in the basal state after treatment with tolgomycin, FCCP, and rotenone/antimycin A. Then, their basal and maximal respirations were calculated. Their ATP production was calculated by subtracting the minimum rate measured after oligomycin injection from the final rate measured in the basal state. The protein content was normalized.

**Statistical analysis**

Statistical analyses were performed using GraphPad Prism 8. The results are depicted as the mean ± SD, and the statistical tests are indicated in the figure legends.

**Result**


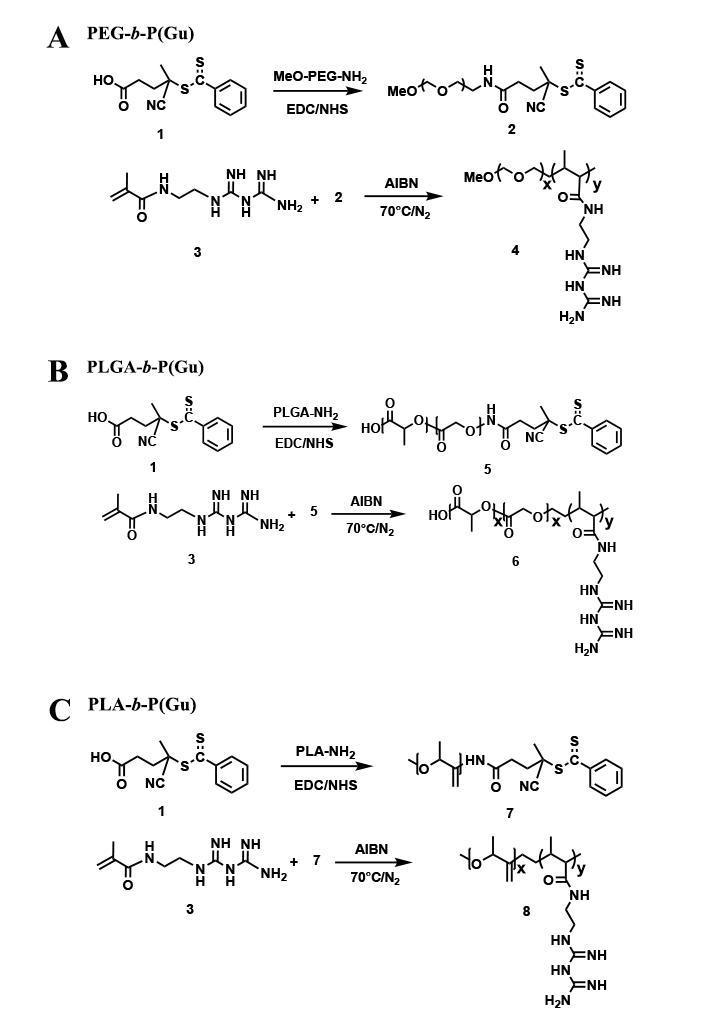


Fig. S1. Synthesis procedure for (A) PEG-***b***-P(Gu), (B) PLGA-***b***-P(Gu), and (C) PLA-***b***-P(Gu).


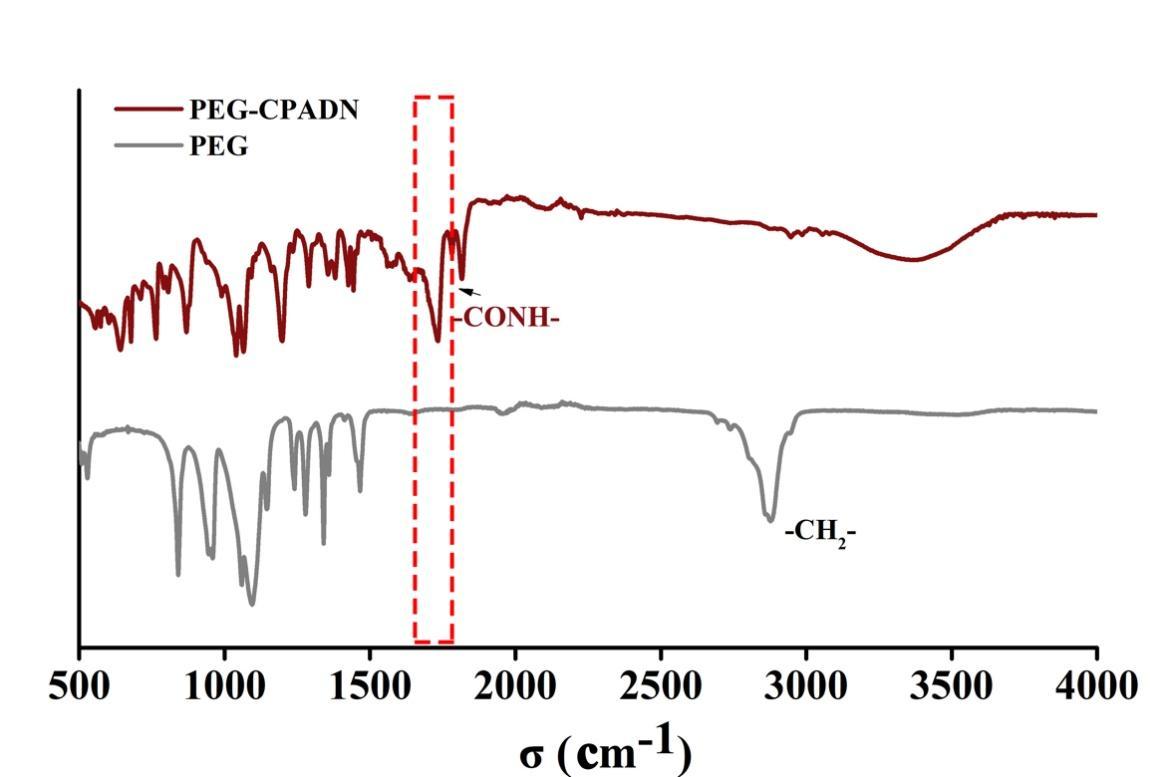


Fig. S2. Infrared spectroscopy (IR) spectra of MeO-PEG_5000_-CPADN (red) and MeO-PEG_5000_-COOH (gray).


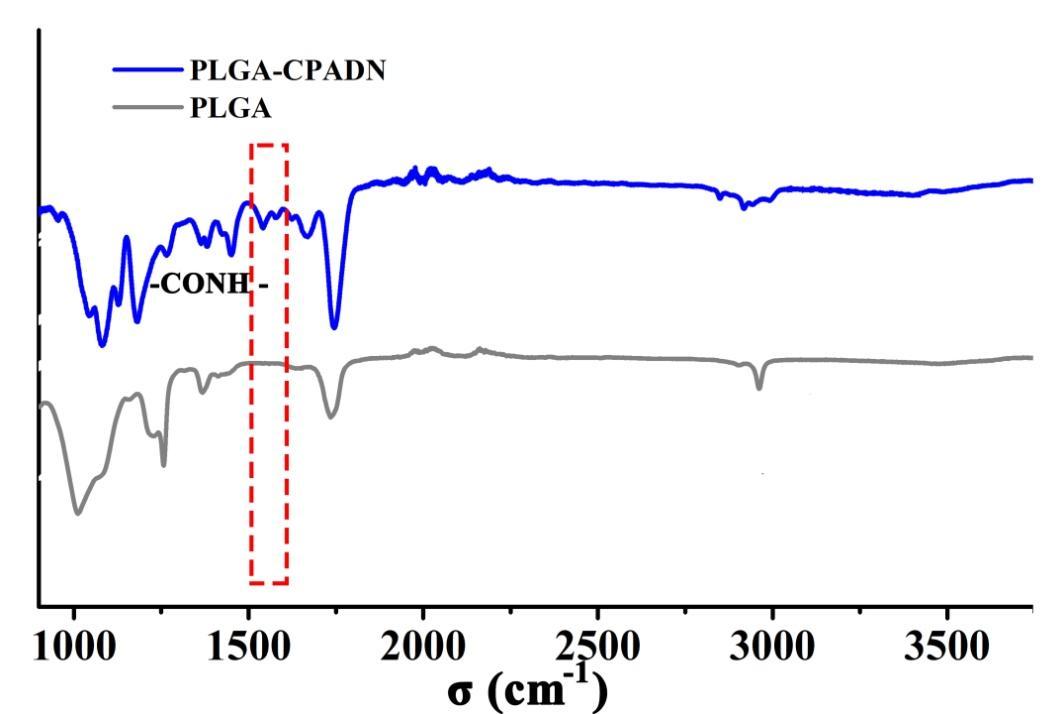


Fig. S3. IR spectra of PLGA_5000_-CPADN (blue) and PLGA_5000_-COOH (gray).


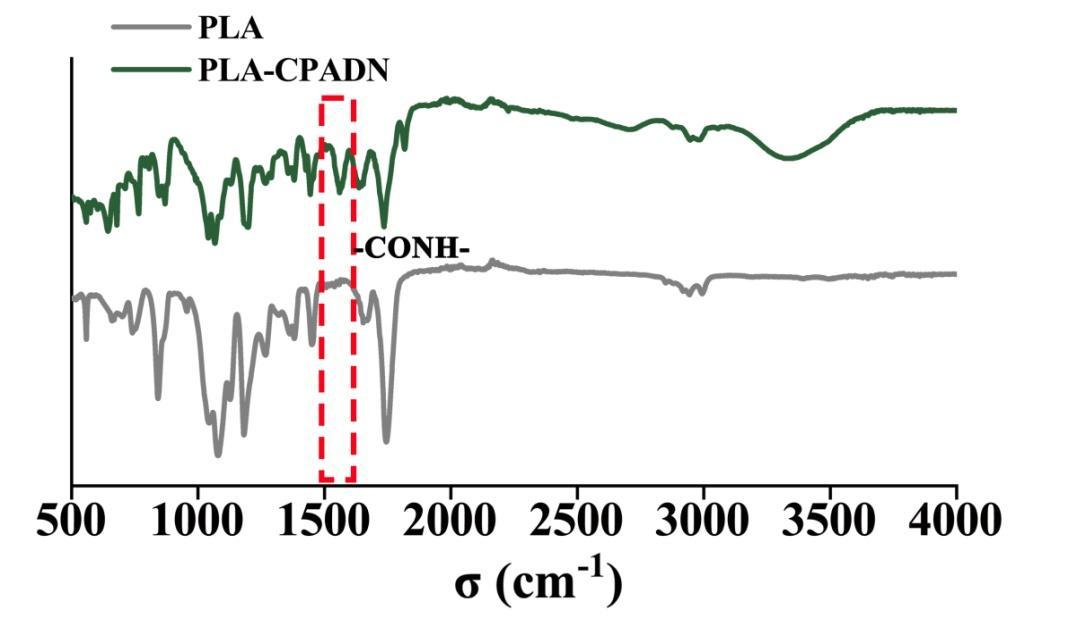


Fig. S4. IR spectra of PLA_5000_-CPADN (green) and PLA_5000_-COOH (gray).


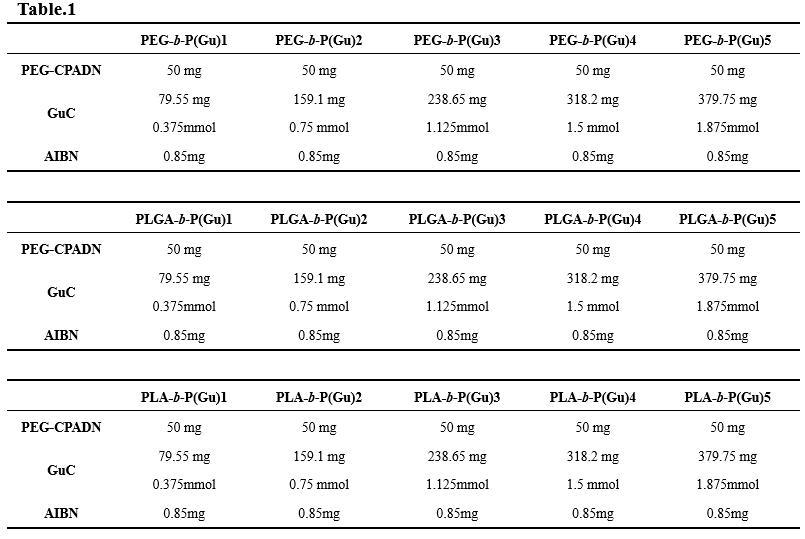


Fig. S5. Feeding ratio of PGNIs.


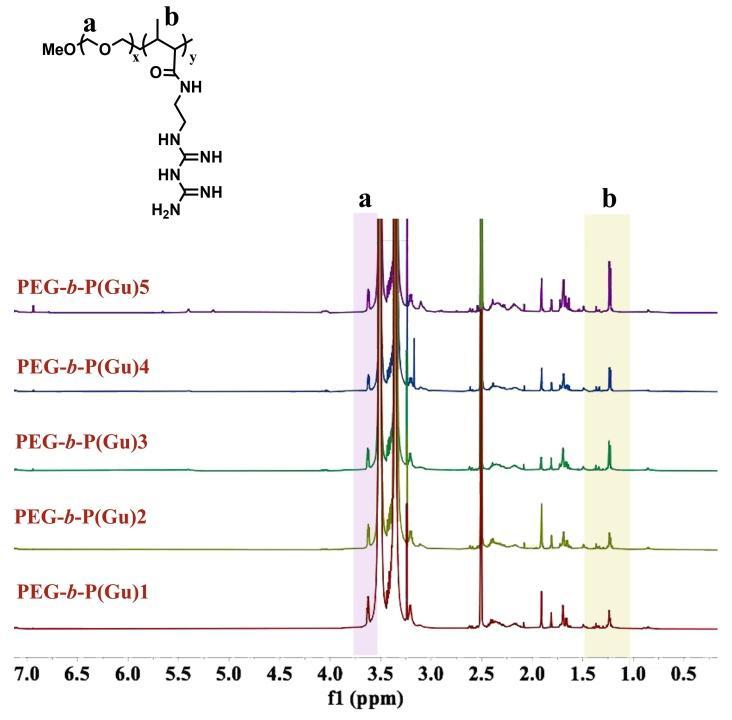


Fig. S6. ^1^H NMR spectrum of PEG-***b***-P(Gu) (C₂D₆SO, 600 MHz).


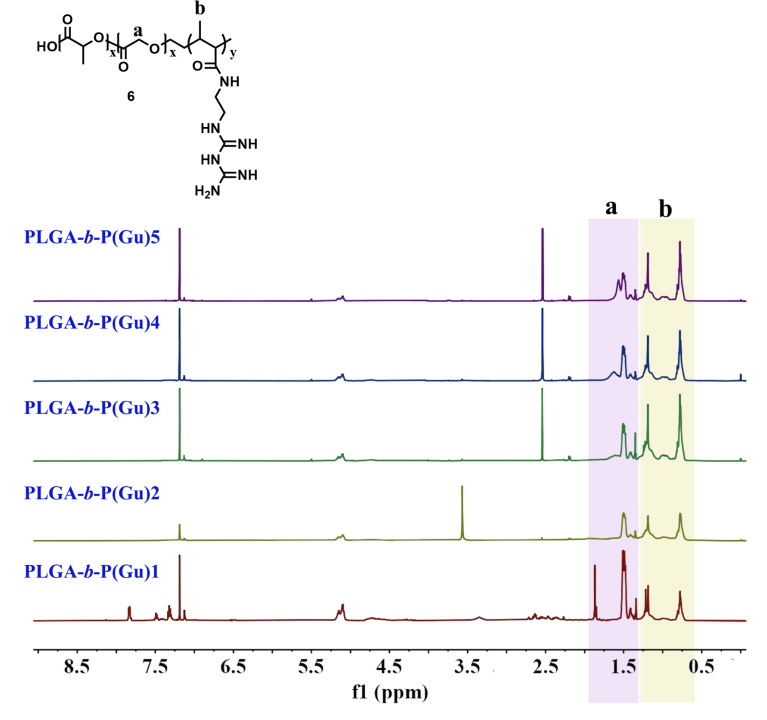


Fig. S7. ^1^H NMR spectrum of PLGA-***b***-P(Gu) (CD_3_Cl, 600 MHz).


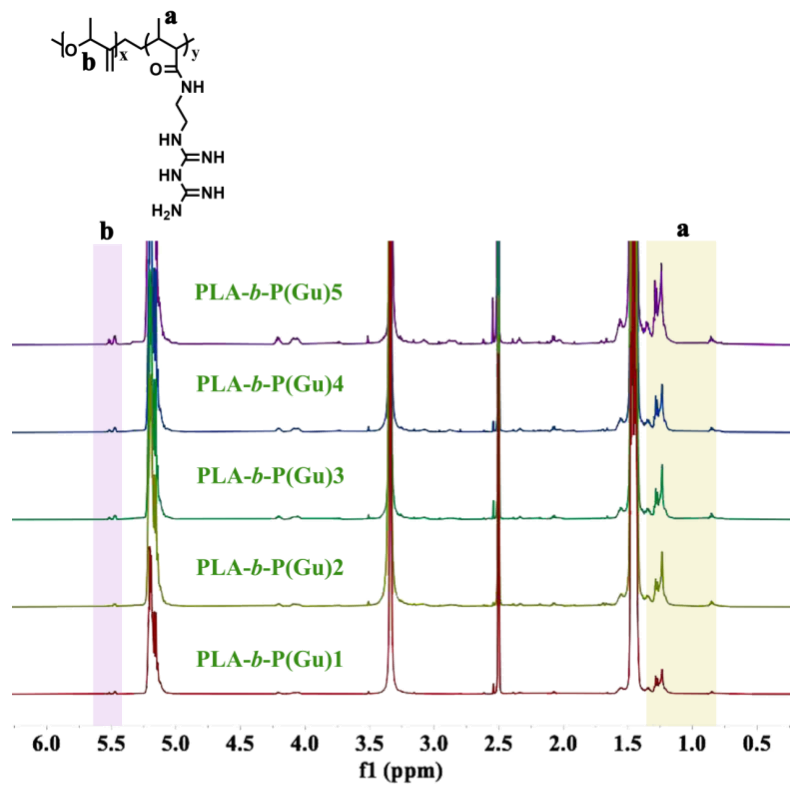


Fig. S8. ^1^H NMR spectrum of PLA-***b***-P(Gu) (C₂D₆SO 600 MHz).


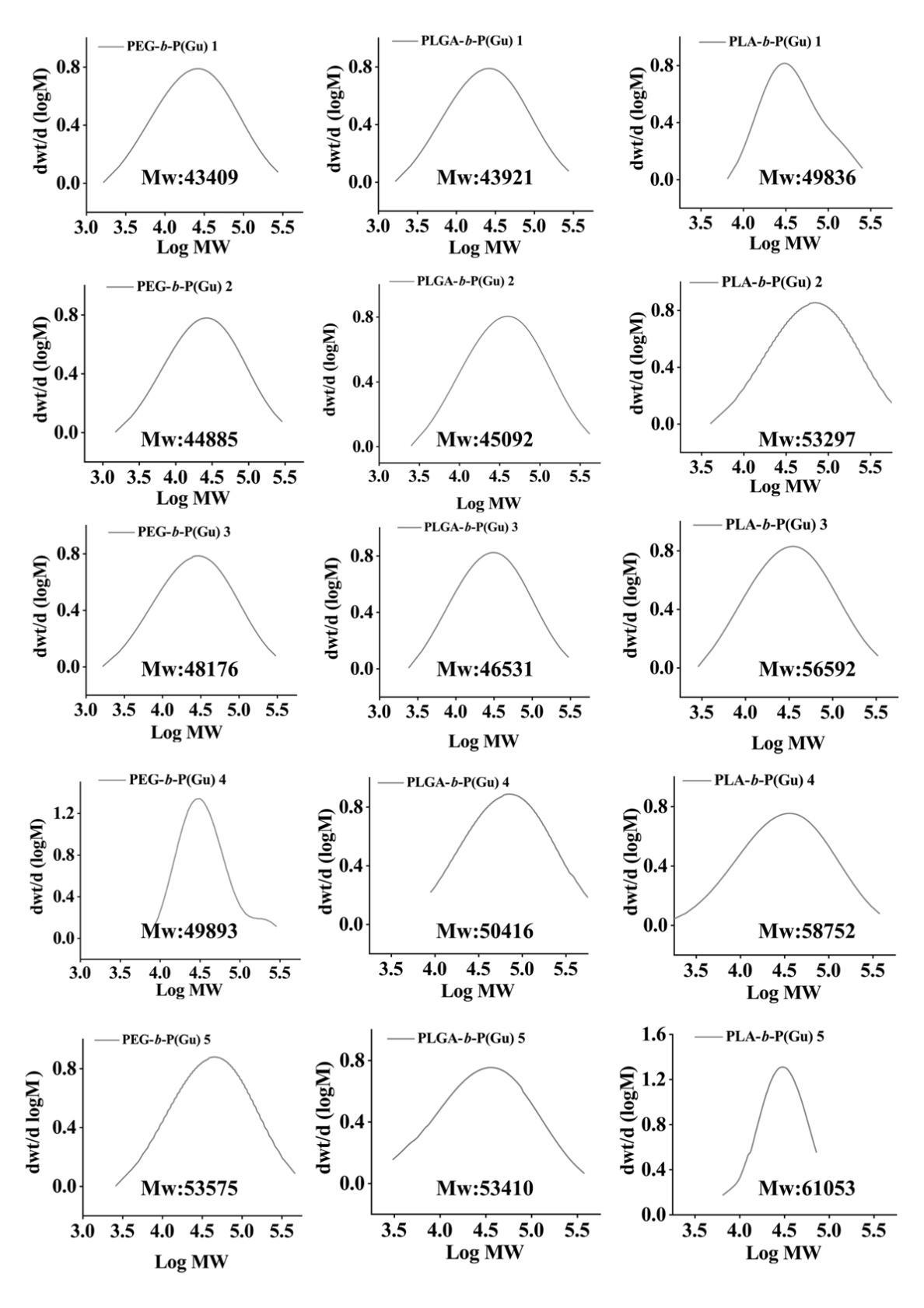


Fig. S9. Gel permeation chromatography (GPC) image of PEG-***b***-P(Gu) 1-5, PLGA-***b***-P(Gu) 1-5, and PLA-***b***-P(Gu) 1-5.


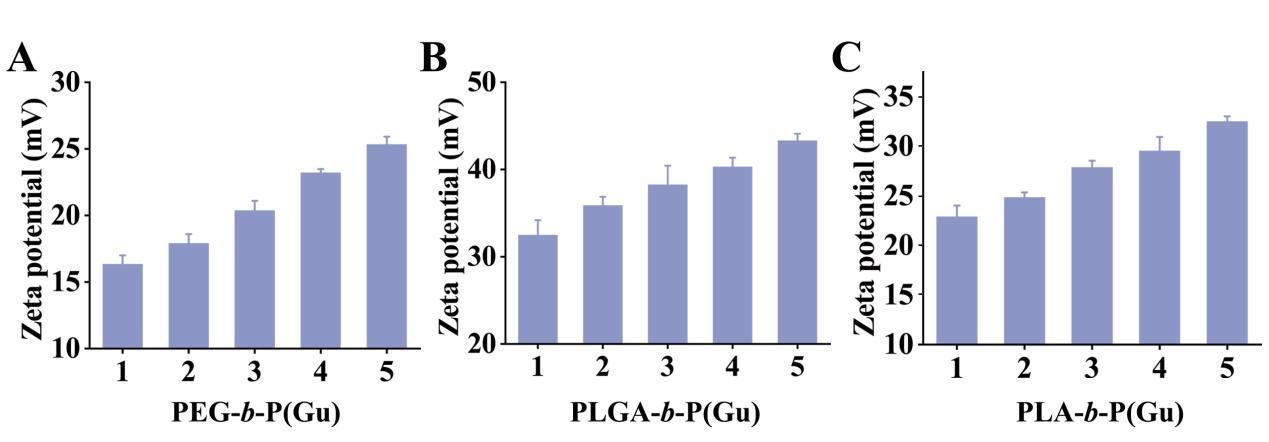


Fig. S10. Zeta potential of (A) PEG-***b***-P(Gu) 1-5, (B) PLGA-***b***-P(Gu) 1-5, and (C) PLA-***b***-P(Gu) 1-5; bars represent the mean ± SD (n = 3).


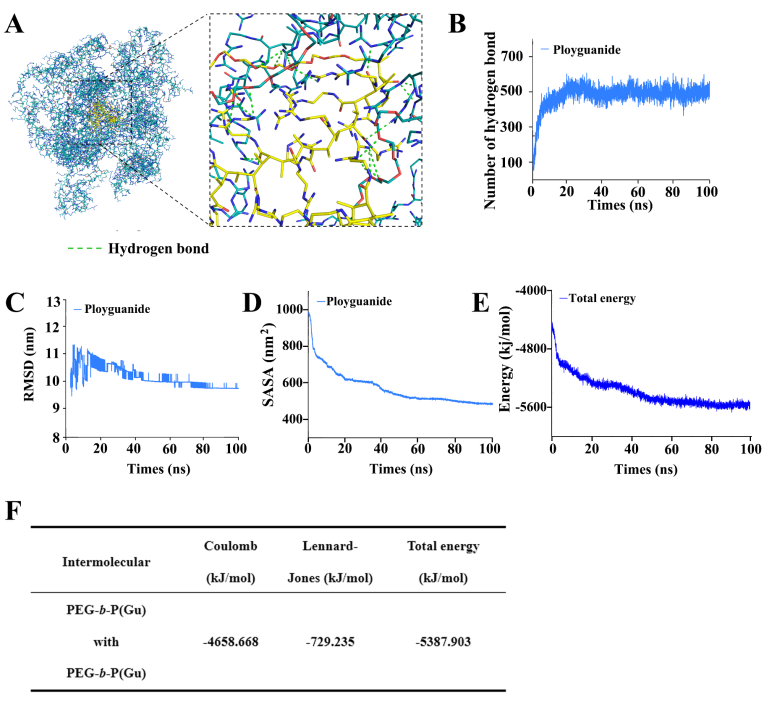


Fig. S11. Molecular dynamics simulation of self-assembly process of PEG-***b***-P(Gu). (A) Interaction pattern diagram between PEG-***b***-P(Gu) molecules in assembly system (green dashed line represents hydrogen bonding). (B) Number of hydrogen bonds, (C) RMSD, (D) solvent accessible surface area, (E) interaction energy, and (F) numerical value between PEG-***b***-P(Gu) molecules changes with simulation time.
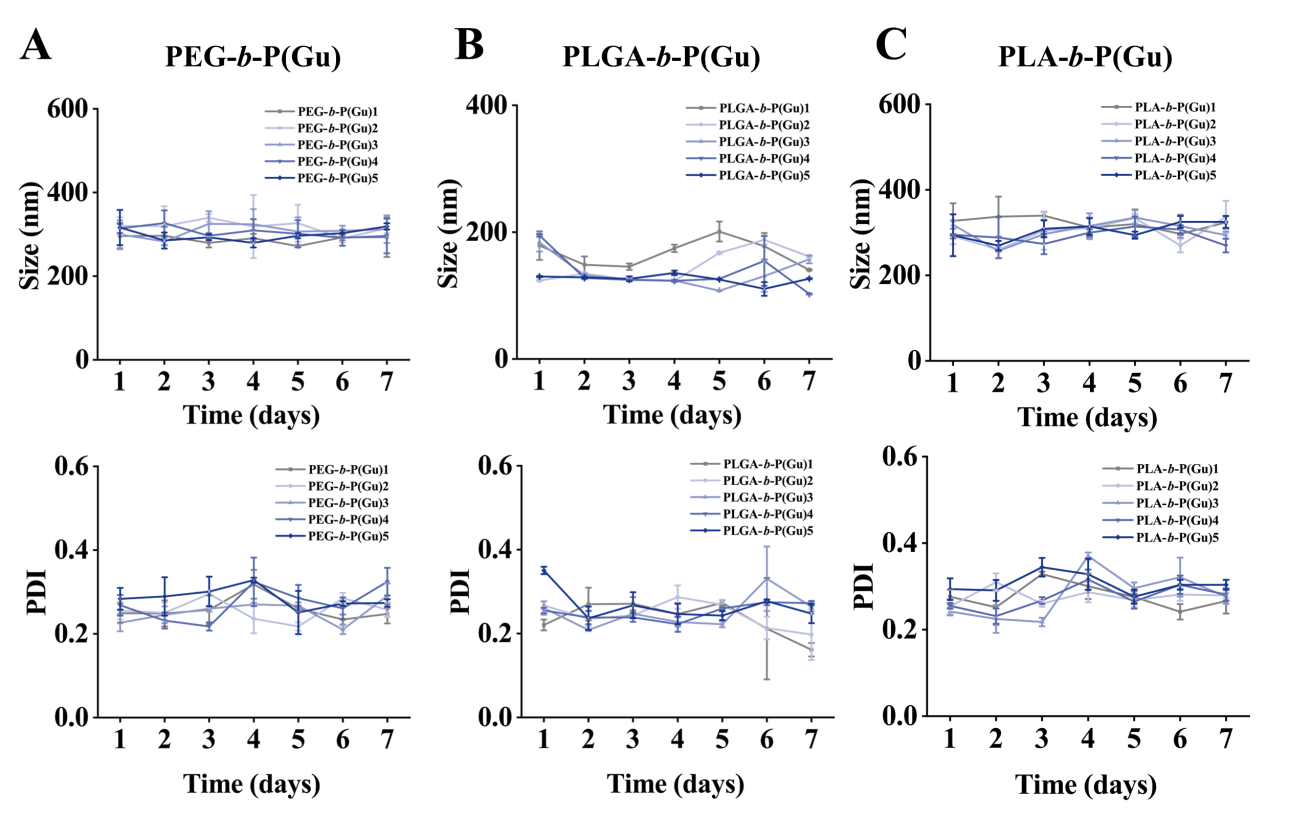


Fig. S12. DLS size and PDI image of PEG-***b***-P(Gu)1-5, PLGA-***b***-P(Gu)1-5, and PLA-***b***-P(Gu)1-5 in 7 days. Bars represent the mean ± SD (n = 3).


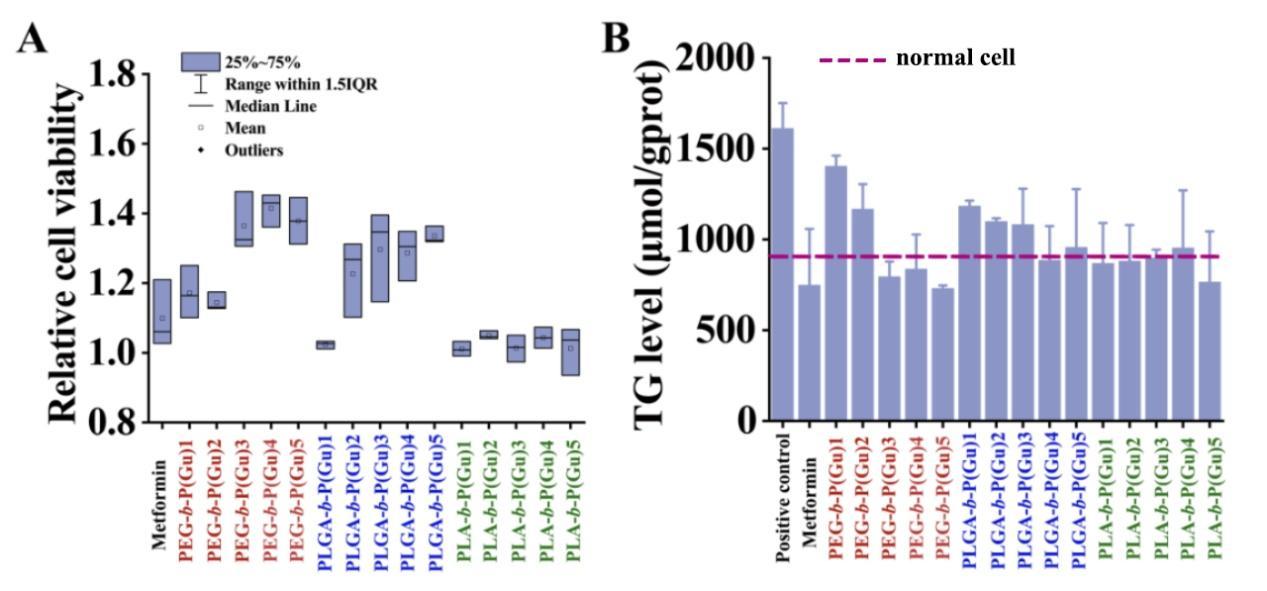


Fig. S13. Quantitative analysis of cell viability and triglyceride levels in HepG2 cells treated with metformin, PEG-***b***-P(Gu)1-5, PLGA-***b***-P(Gu)1-5, or PLA-***b***-P(Gu)1-5; bars represent the mean ± SD (n = 3).


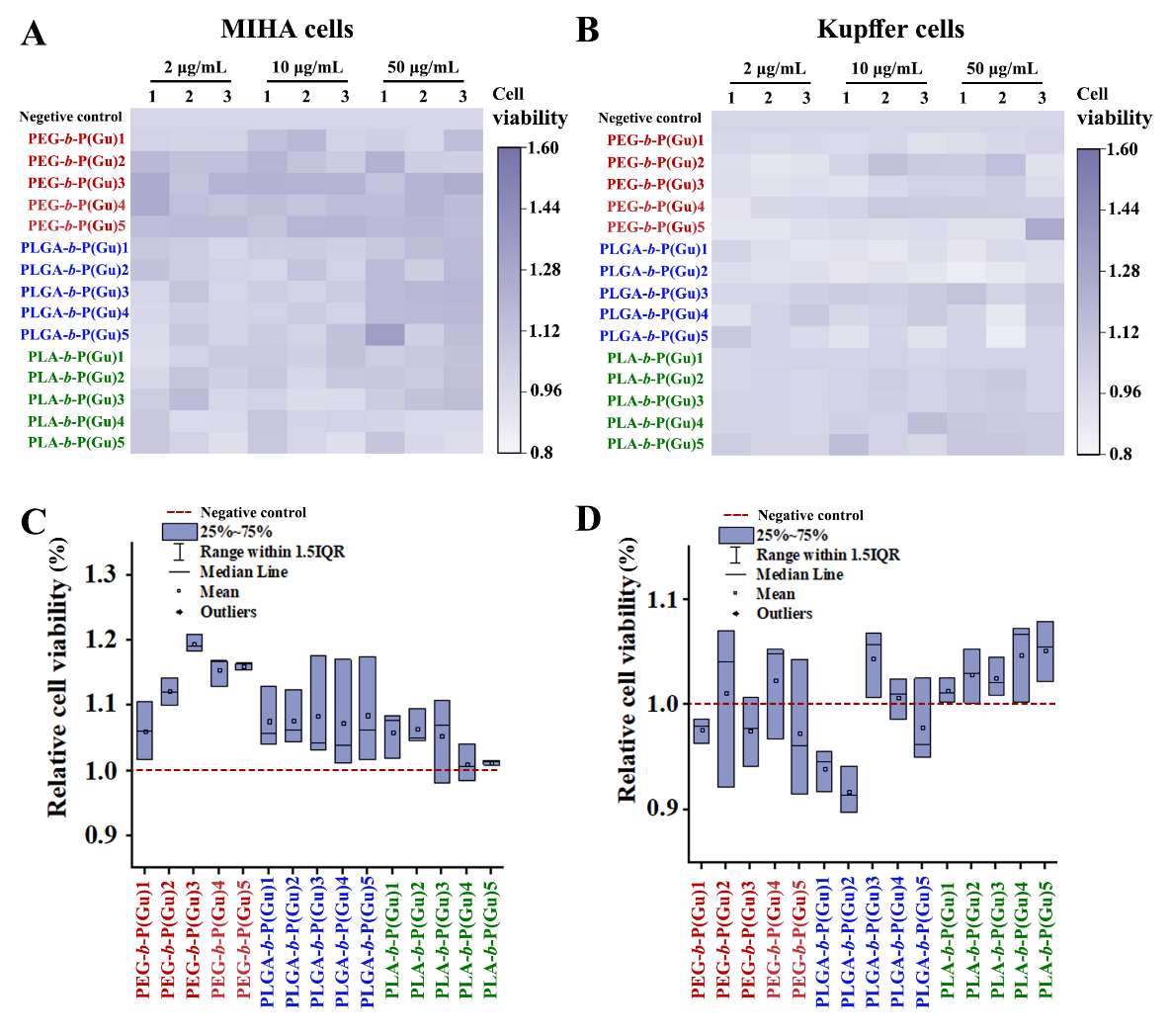


Fig. S14. Relative cell viability and quantitative analysis of MIHA cells (normal hepatocytes) and Kupffer cells treated with PEG-***b***-P(Gu)1-5, PLGA-***b***-P(Gu)1-5, and PLA-***b***-P(Gu)1-5 in different concentrations.


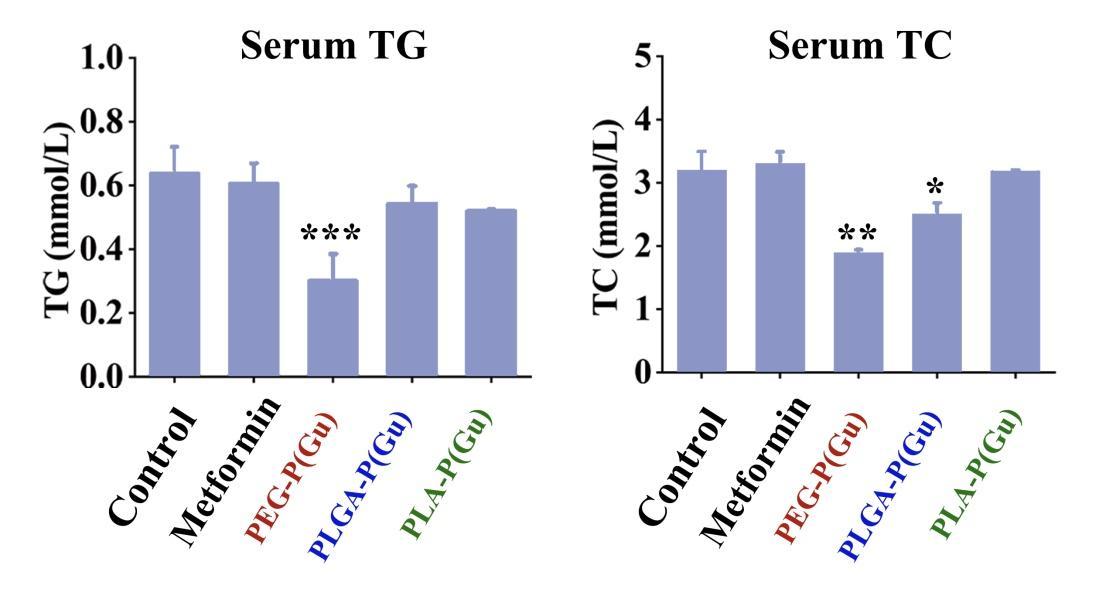


Fig. S15. Total triglycerides (TG) and total cholesterol (TC) levels in serum after 16 weeks of treatment with metformin, PEG-***b***-P(Gu), PLGA-***b***-P(Gu), or PLA-***b***-P(Gu). ***P < 0.001; **P< 0.01; *P < 0.05; bars represent the mean ± SD (n = 6). The P-values are calculated using one-way ANOVA.


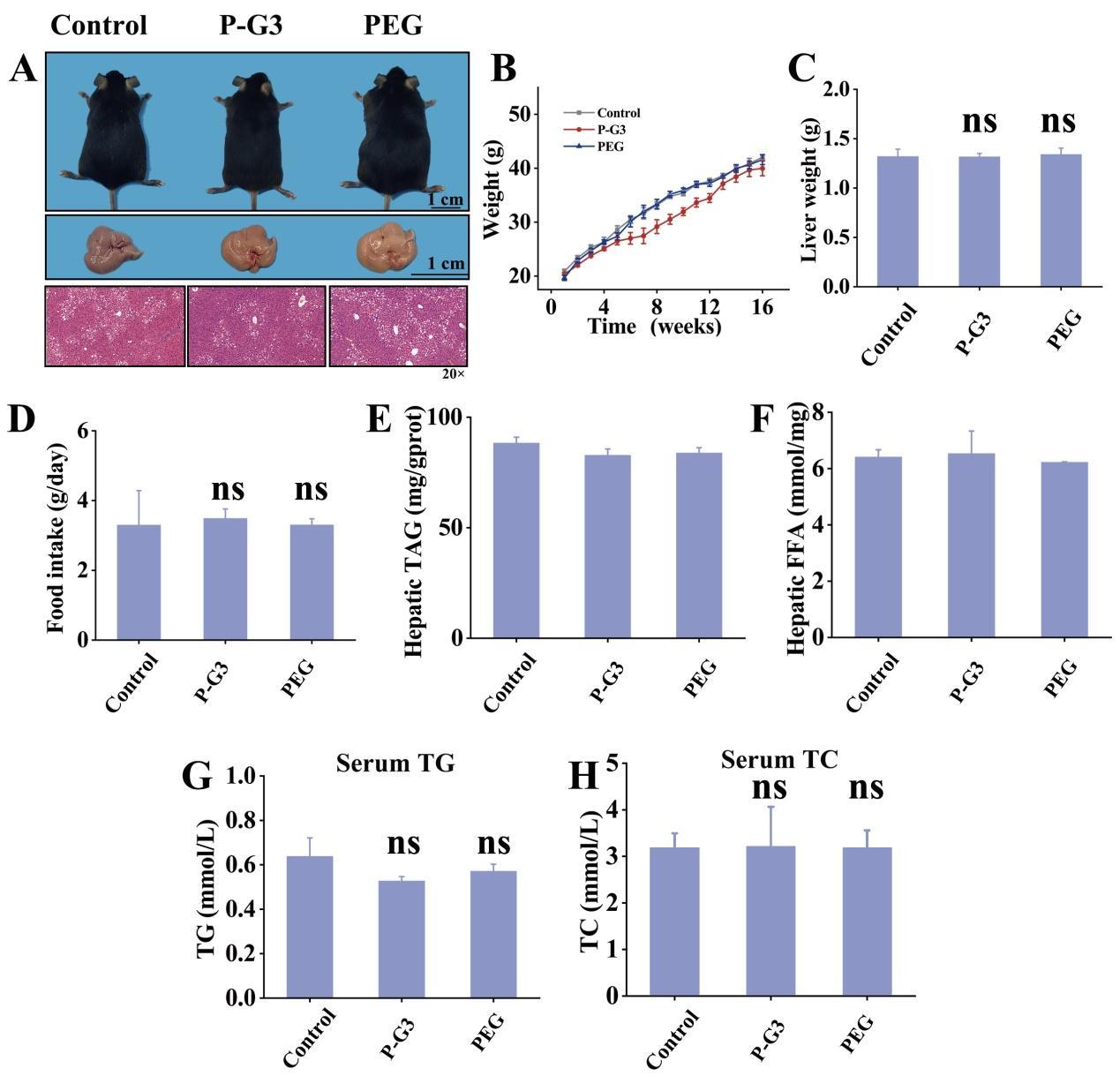


Fig. S16. (A) H&E staining images of C57BL/6J mice whole-body and liver after treatment with P-G3 and PEG. (B) Body weight, (C) liver weight, and (D) food intake of P-G3 and PEG group mice. (E) Hepatic TG, (F) hepatic FFA, (G) serum TG, and (H) serum TC after administration with P-G3 and PEG. ns ＞ 0.05; bars represent the mean ± SD (n = 6). The P-values are calculated using one-way ANOVA.


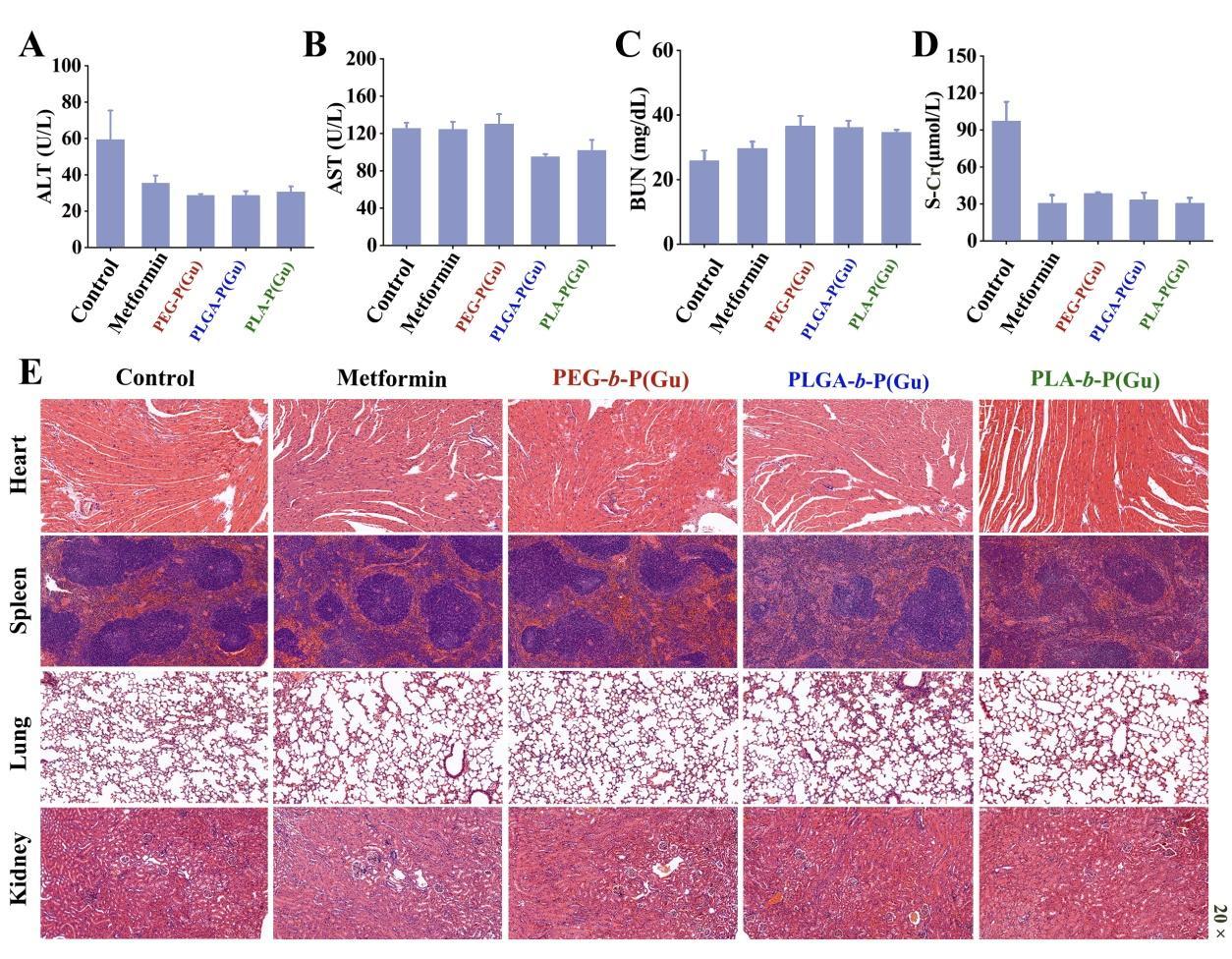


Fig. S17. Levels of typical hematological parameters of mice, including (A) ALT, (B) AST, (C) BUN, and (D) S-cr of mice treated with metformin, PEG-***b***-P(Gu), PLGA-***b***-P(Gu), and PLA-***b***-P(Gu). Bars represent the mean ± SD (n = 6). (E) H&E staining images of major organs collected from mice treated with PEG-***b***-P(Gu), PLGA-***b***-P(Gu), and PLA-***b***-P(Gu) and from the control group.


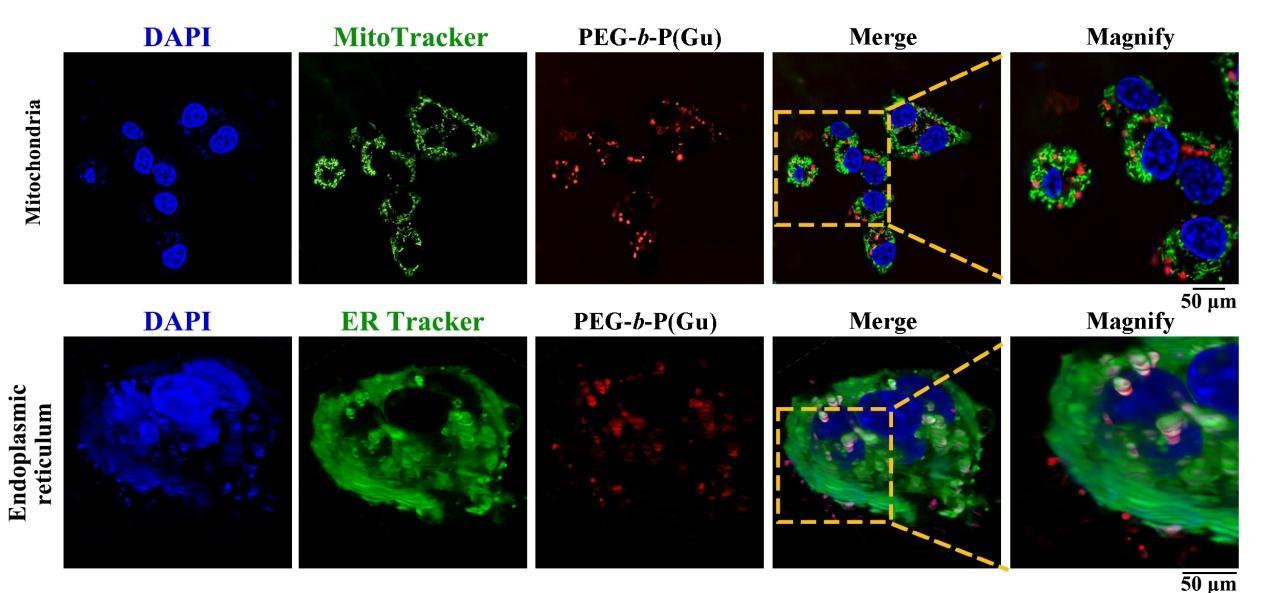


Fig. S18. CLSM of PEG-***b***-P(Gu) along with other organelles in HepG2 cells. Blue: nucleus, green: mitochondria or endoplasmic reticulum, red: PEG-***b***-P(Gu). Scale bar: 50 μm.


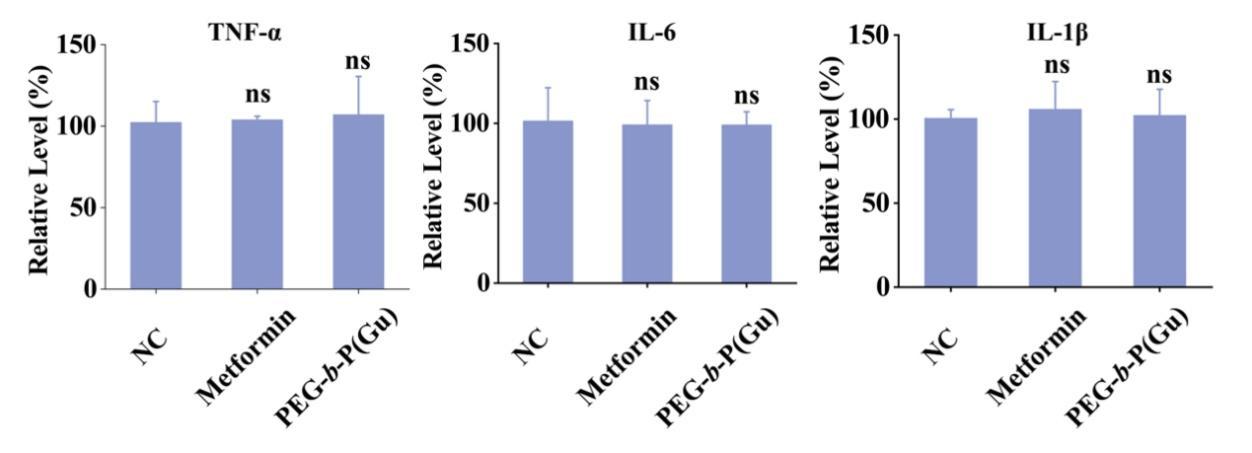


Fig. S19. qPCR analysis of the gene expression of inflammatory markers in mouse liver, including IL-6, IL-1β, and TNF-α. ns ＞ 0.05; bars represent the mean ± SD (n = 6). The P-values are calculated using one-way ANOVA.


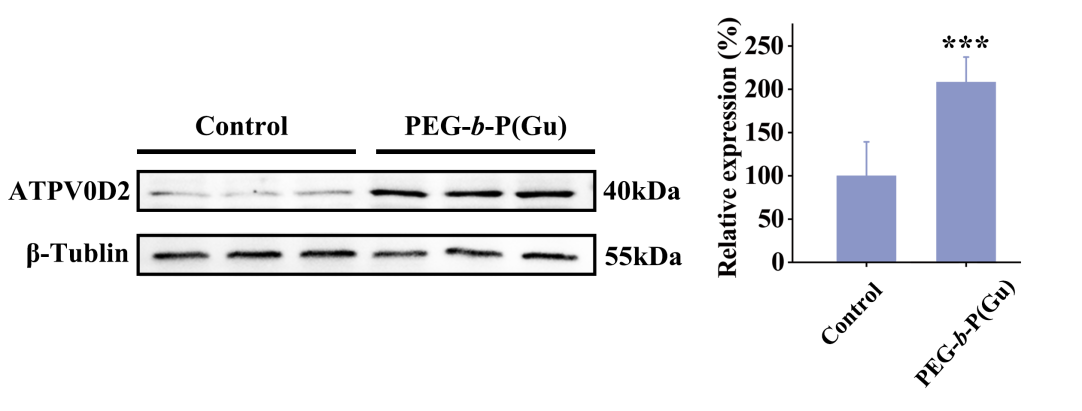


Fig. S20. Expression of ATPV0D2 protein, and their quantitative data in mouse liver (n = 6). ***P < 0.001; **P< 0.01; *P < 0.05; bars represent the mean ± SD (n = 6).


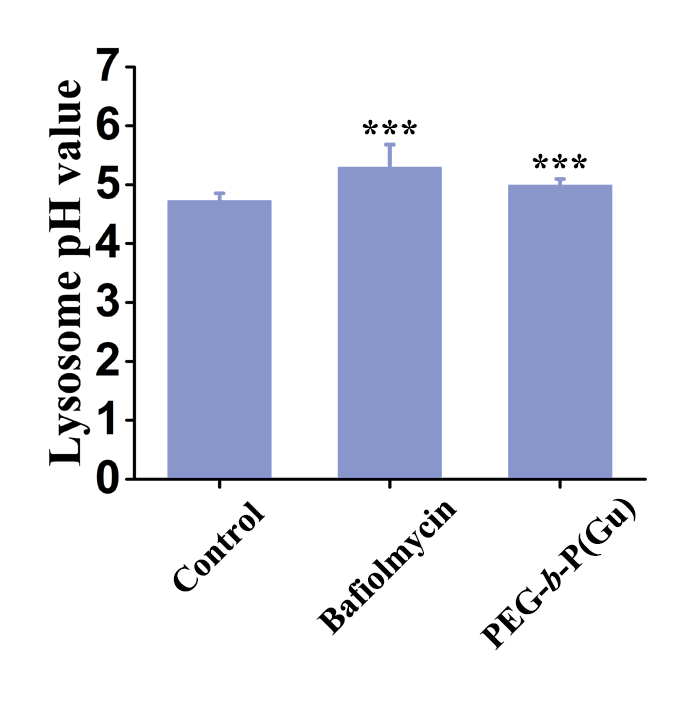


Fig. S21. pH value of lysosomes in HepG2 after treatment with V-ATPase inhibitor bafilomycin and PEG-***b***-P(Gu) (n = 6). ***P < 0.001; **P< 0.01; *P < 0.05; Control means palmitic acid loading of HepG2 cells. Bars represent the mean ± SD (n = 6). The P-values are calculated using one-way ANOVA.


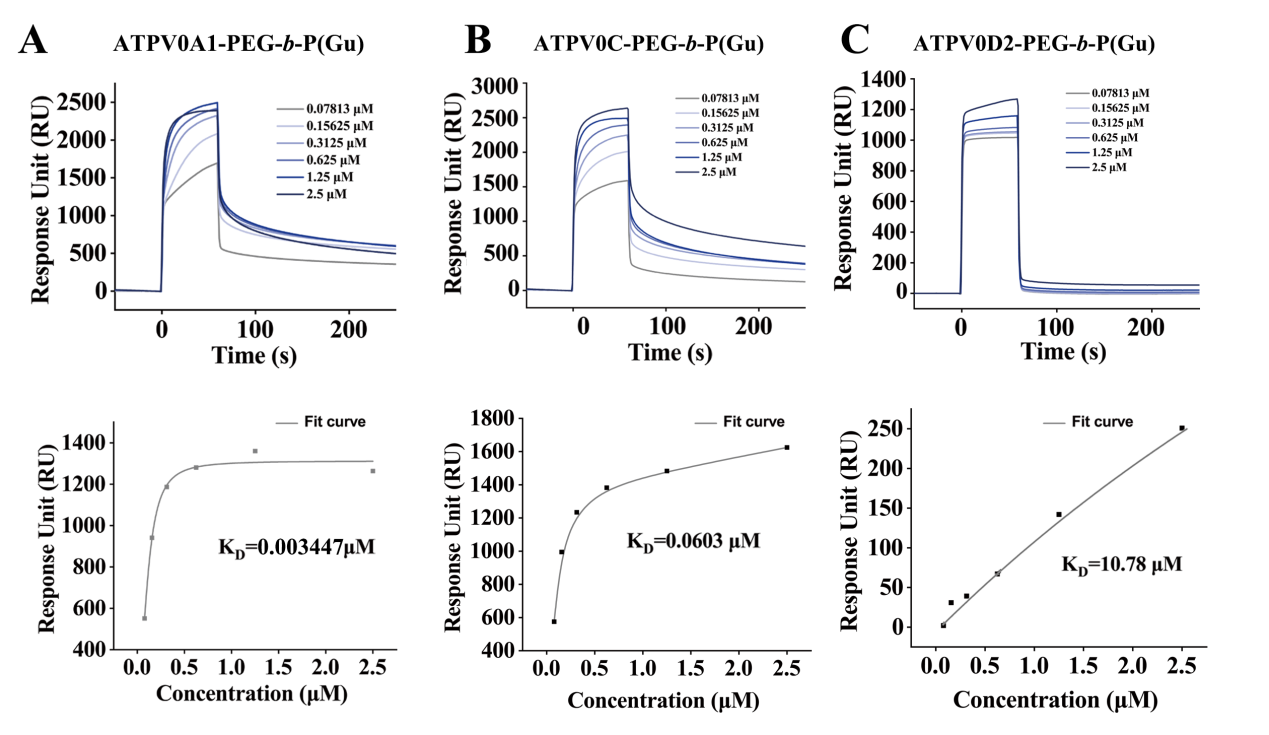


Fig. S22. Surface plasmon resonance (SPR) of PEG-***b***-P(Gu) between V-ATPase subunit ATPV0A1, ATPV0C, and ATPV0D2.


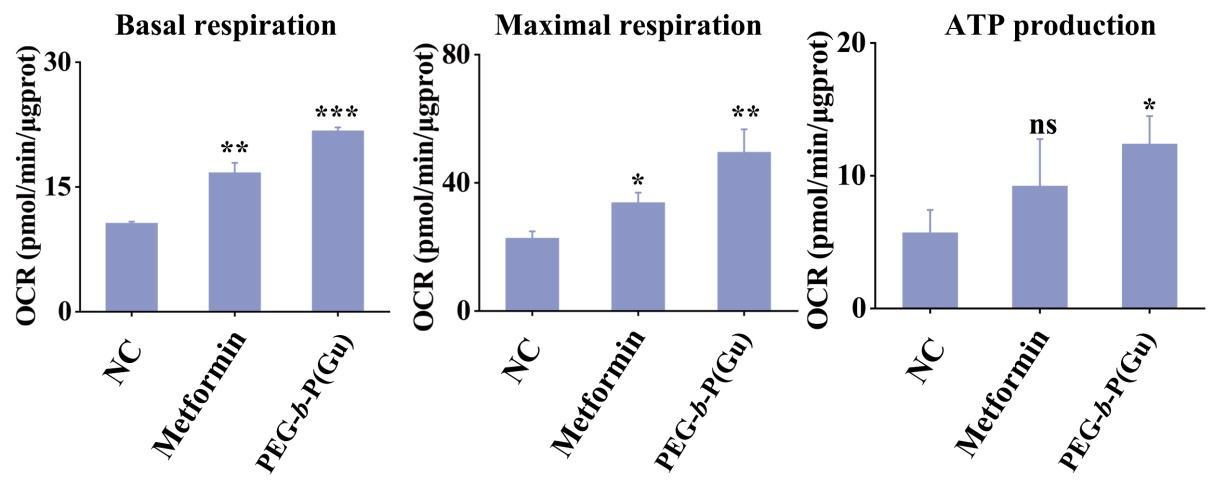


Fig. S23. Statistical analysis of oxygen consumption rate in HepG2 cells. ***P < 0.001; **P< 0.01; *P < 0.05; bars represent the mean ± SD (n = 3). The P-values are calculated using one-way ANOVA.


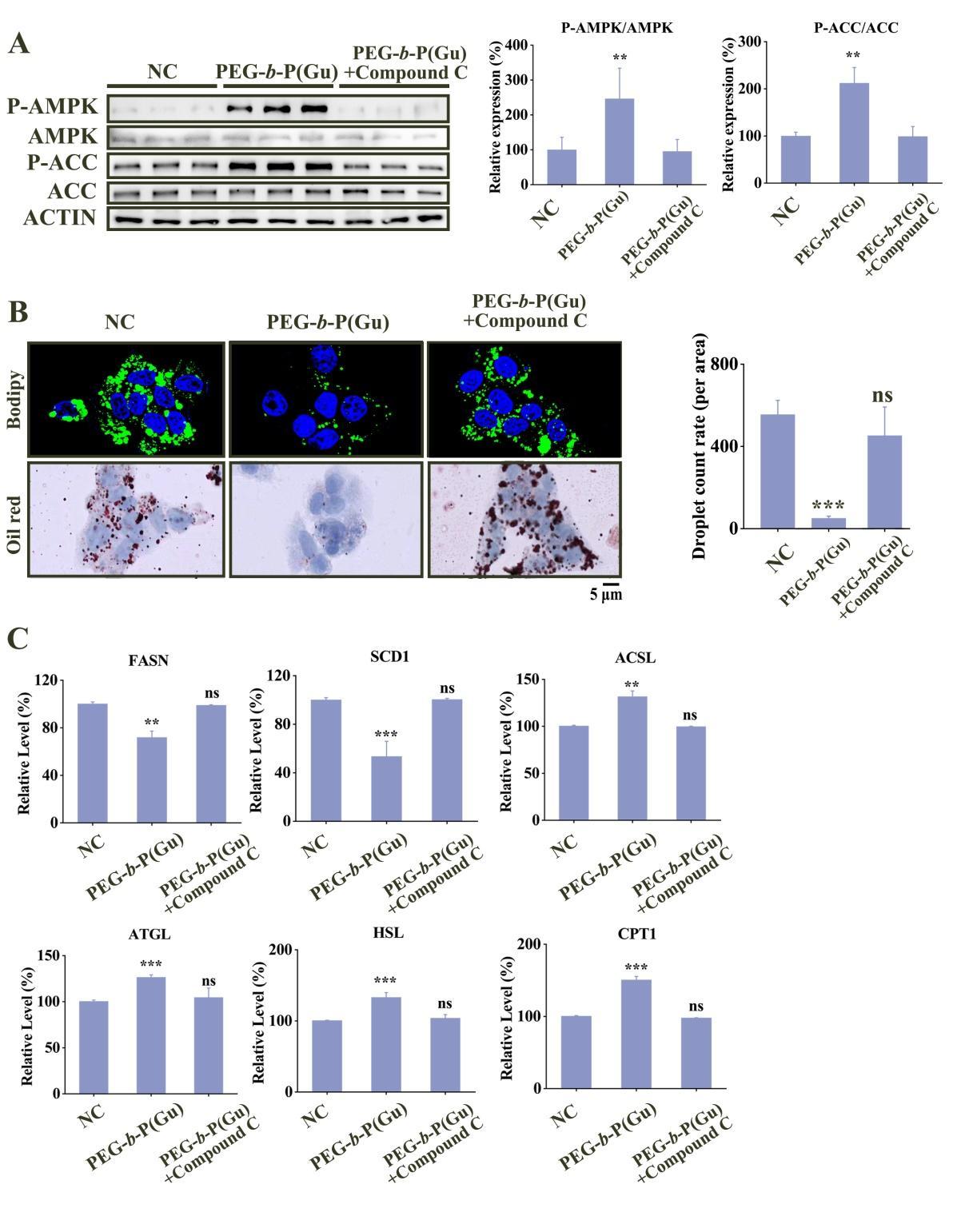


Fig. S24. Interruption of the effect of PEG-***b***-P(Gu) in HepG2 cells by AMPK inhibitors (compound C). (A) Expression and phosphorylation levels of AMPK and ACC protein, and their quantitative data after treatment with PEG-***b***-P(Gu) and PEG-***b***-P(Gu)/Compound C. ***P < 0.001; **P< 0.01; *P < 0.05; statistical analysis was performed using one-way ANOVA; bars represent the mean ± SD (n = 3). (B) Bodipy and Oil Red O staining and lipid content rate of HepG2 cells treated with PEG-***b***-P(Gu) and PEG-***b***-P(Gu)/Compound C. Scale bar: 5 μm. ***P < 0.001; **P< 0.01; *P < 0.05; statistical analysis was performed using one-way ANOVA; bars represent the mean ± SD (n = 3). (C) qPCR analysis of the gene expression of fatty acid synthesis, lipolysis, and β-oxidation markers after treatment with PEG-***b***-P(Gu) and PEG-***b***-P(Gu)/Compound C. Bars represent the mean ± SD (n = 3); ***P < 0.001; **P< 0.01; *P < 0.05; statistical analysis was performed using one-way ANOVA.


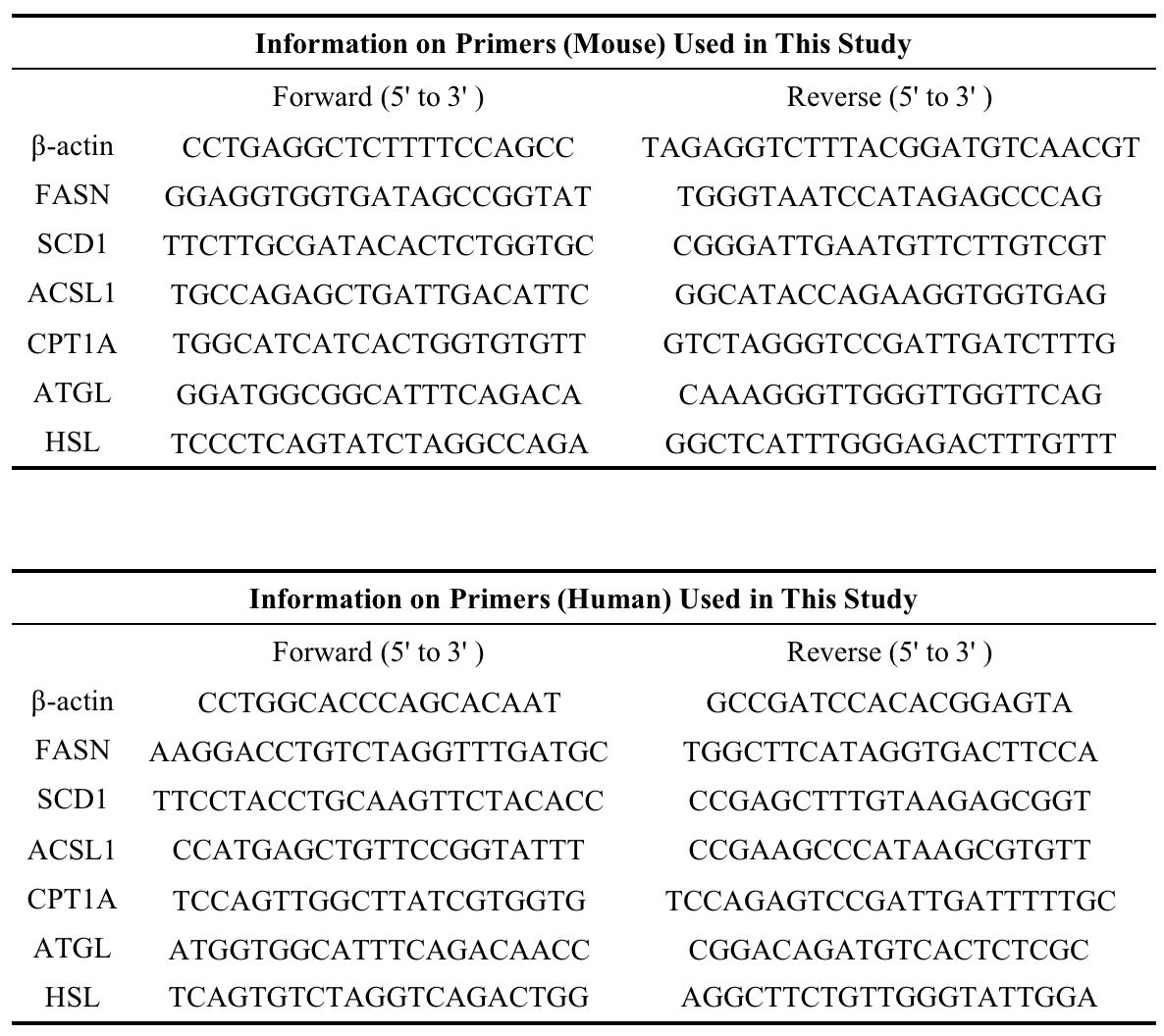


Fig. S25. Sequences of primers used in this study.

**Reference**

[1] F. Andreata, C. BlÃ©riot, P. Di Lucia, G. De Simone, V. Fumagalli, X. Ficht, C. G. Beccaria, M. Kuka, F. Ginhoux, M. Iannacone*, STAR protocol***s 20**2**1***,* 2 (4), 100831.
